# Subversion of natural killer cell responses by a cytomegalovirus-encoded soluble CD48 decoy receptor

**DOI:** 10.1371/journal.ppat.1007658

**Published:** 2019-04-04

**Authors:** Pablo Martínez-Vicente, Domènec Farré, Carolina Sánchez, Antonio Alcamí, Pablo Engel, Ana Angulo

**Affiliations:** 1 Immunology Unit, Department of Biomedical Sciences, Faculty of Medicine and Health Sciences, University of Barcelona, Barcelona, Spain; 2 Centro de Biología Molecular Severo Ochoa (Consejo Superior de Investigaciones Científicas y Universidad Autónoma de Madrid), Madrid, Spain; 3 Institut d’Investigacions Biomèdiques August Pi i Sunyer, Barcelona, Spain; Harvard Medical School, UNITED STATES

## Abstract

Throughout evolution, cytomegaloviruses (CMVs) have been capturing genes from their hosts, employing the derived proteins to evade host immune defenses. We have recently reported the presence of a number of *CD48* homologs (vCD48s) encoded by different pathogenic viruses, including several CMVs. However, their properties and biological relevance remain as yet unexplored. CD48, a cosignaling molecule expressed on the surface of most hematopoietic cells, modulates the function of natural killer (NK) and other cytotoxic cells by binding to its natural ligand 2B4 (CD244). Here, we have characterized A43, the vCD48 exhibiting the highest amino acid sequence identity with host CD48. A43, which is encoded by owl monkey CMV, is a soluble molecule released from the cell after being proteolytically processed through its membrane proximal region. *A43* is expressed with immediate-early kinetics, yielding a protein that is rapidly detected in the supernatant of infected cells. Remarkably, surface plasmon resonance assays revealed that this viral protein binds to host 2B4 with high affinity and slow dissociation rates. We demonstrate that soluble A43 is capable to abrogate host CD48:2B4 interactions. Moreover, A43 strongly binds to human 2B4 and prevents 2B4-mediated NK-cell adhesion to target cells, therefore reducing the formation of conjugates and the establishment of immunological synapses between human NK cells and CD48-expressing target cells. Furthermore, in the presence of this viral protein, 2B4-mediated cytotoxicity and IFN-γ production by NK cells are severely impaired. In summary, we propose that A43 may serve as a functional soluble CD48 decoy receptor by binding and masking 2B4, thereby impeding effective NK cell immune control during viral infections. Thus, our findings provide a novel example of the immune evasion strategies developed by viruses.

## Author summary

In order to evade detection and destruction by cytotoxic lymphocytes and successfully persist within their hosts, cytomegalovirus (CMVs) have evolved a number of genes dedicated to block immune recognition. Certain CMVs and other large DNA viruses encode homologs of the cell-surface molecule CD48, a ligand of the 2B4 receptor involved in regulating the function of cytotoxic lymphocytes. Here, we have investigated for the first time the immunomodulatory potential of one of these viral molecules. We show that A43, a CD48 homolog encoded by owl monkey CMV, is a soluble molecule that exhibits exceptional binding kinetics for 2B4, and is furthermore capable of blocking the interaction with its counter-receptor CD48. Moreover, we reveal how this viral protein interferes with human NK cell-mediated cytotoxicity by inhibiting the immune synapse between human NK cells and target cells. Thus, these findings not only underscore the importance of 2B4-mediated immune responses in controlling CMV infections, but also unveil the shedding of a virally-encoded soluble variant of CD48 as a new viral counteract mechanism for subverting immune surveillance.

## Introduction

Natural killer (NK) cells are circulating lymphocytes that play a pivotal role in the rapid recognition and control of viral infections. NK functions are regulated by a repertoire of specific receptors that, upon engagement with their respective ligands on target cells, transmit stimulatory or inhibitory signals [1]. The net balance of activating/inhibitory signals determines whether the NK cell will initiate its cytolytic activity through the degranulation of specialized secretory lysosomes into the immune synapse, ultimately causing the destruction of the target cell. One such receptor is 2B4 (or CD244), a member of the signaling lymphocyte activation molecule (SLAM) family of the immunoglobulin (Ig) superfamily [2]. In human NK cells, 2B4 predominantly provides co-stimulatory signals, activating NK cytotoxicity and cytokine production [3]. 2B4 interacts with CD48, another member of the SLAM family that is broadly expressed on the surface of most hematopoietic cells [4–6]. Both receptors contain an ectodomain composed of an N-terminal Ig membrane-distal variable (IgV) domain followed by an Ig constant-2-set domain, characterized by conserved cysteines. However, while CD48 is a glycosyl-phosphatidylinositol (GPI)-anchored protein, 2B4 is a type I transmembrane molecule that contains four copies of the immune receptor tyrosine-based switch motif (ITSM) in its cytoplasmic tail [7, 8]. 2B4 engagement by CD48 occurs through their N-terminal IgV domains, resulting in the recruitment of specific adaptor molecules by the ITSM motifs followed by signaling transduction events that ultimately modulate immune responses [9]. In addition to NK cells, 2B4 is expressed at lower levels on other cytotoxic cells, including CD8^+^ T cells, γδ T cells, basophils and eosinophils [10, 11]. Therefore, the 2B4:CD48 interaction also contributes to the regulation of additional aspects of the innate and adaptive immune responses.

NK cells are crucial for the successful control of infections by cytomegaloviruses (CMVs). Consequently, these pathogens have evolved a wealth of strategies to hinder or abrogate NK functions [12–15]. Most of these strategies are based on mechanisms designed to avoid recognition of infected cells by activating NK cell receptors or to trigger inhibitory NK cell signaling. To this end, within their large and densely packed genomes, CMVs encode multiple immunosubversive proteins, meticulously shaped for these purposes [16, 17]. Some of these immunoevasins are of cellular origin, having been captured from their hosts at different times during their co-evolution [18–20], and often employed to mimic or interfere with the original host function. Given the relevance of 2B4 for the regulation of NK cell activity, it is not surprising that among their immune evasion strategies CMVs have developed mechanisms to counteract CD48:2B4 interactions. In this regard, we have reported that CMVs reduce the expression of the CD48 receptor on the surface of infected target cells [21]. Indeed, our group identified, in murine CMV, the mucin-like m154 protein as the viral molecule responsible for reducing CD48 macrophage surface expression. We showed that m154 targets CD48 for degradation and helps to impair antiviral NK-triggered cell responses, thereby meliorating viral growth *in vivo*. Moreover, we have recently identified several CD48 homologs (vCD48s) among CMVs [22, 23]. In particular, two primate cytomegaloviruses, SMCMV that infects squirrel monkeys and OMCMV that infects owl monkeys, encode copies of *vCD48s*, which arose from a unique gene-capture event in an ancestor CMV genome and subsequent gene duplication episodes. One of these vCD48s, A43, which shows the highest homology to host CD48, is a secreted molecule that preserves ligand-binding properties, being able to interact with host 2B4 [23]. However, to date, the biological significance of A43 and the rest of these vCD48s remains unknown. Thus, the potential of these molecules to function in immune evasion mechanisms warrants further investigation.

Here, we have characterized the OMCMV encoded CD48 homolog A43. We show that this viral protein can act as a soluble decoy CD48 receptor, protecting cells against NK cell-mediated cytotoxicity. By binding with high affinity to 2B4, this viral protein is capable of preventing CD48:2B4 interactions. In addition, we present that A43 limits human NK cell adhesion to CD48-expressing target cells, inhibiting the formation of the NK cell immunological synapse. Thus, our findings point to a novel class of immunoevasins.

## Results

### *A43* is an immediate early (IE) gene encoding a protein shed from virally infected cells

**Fig 1:**
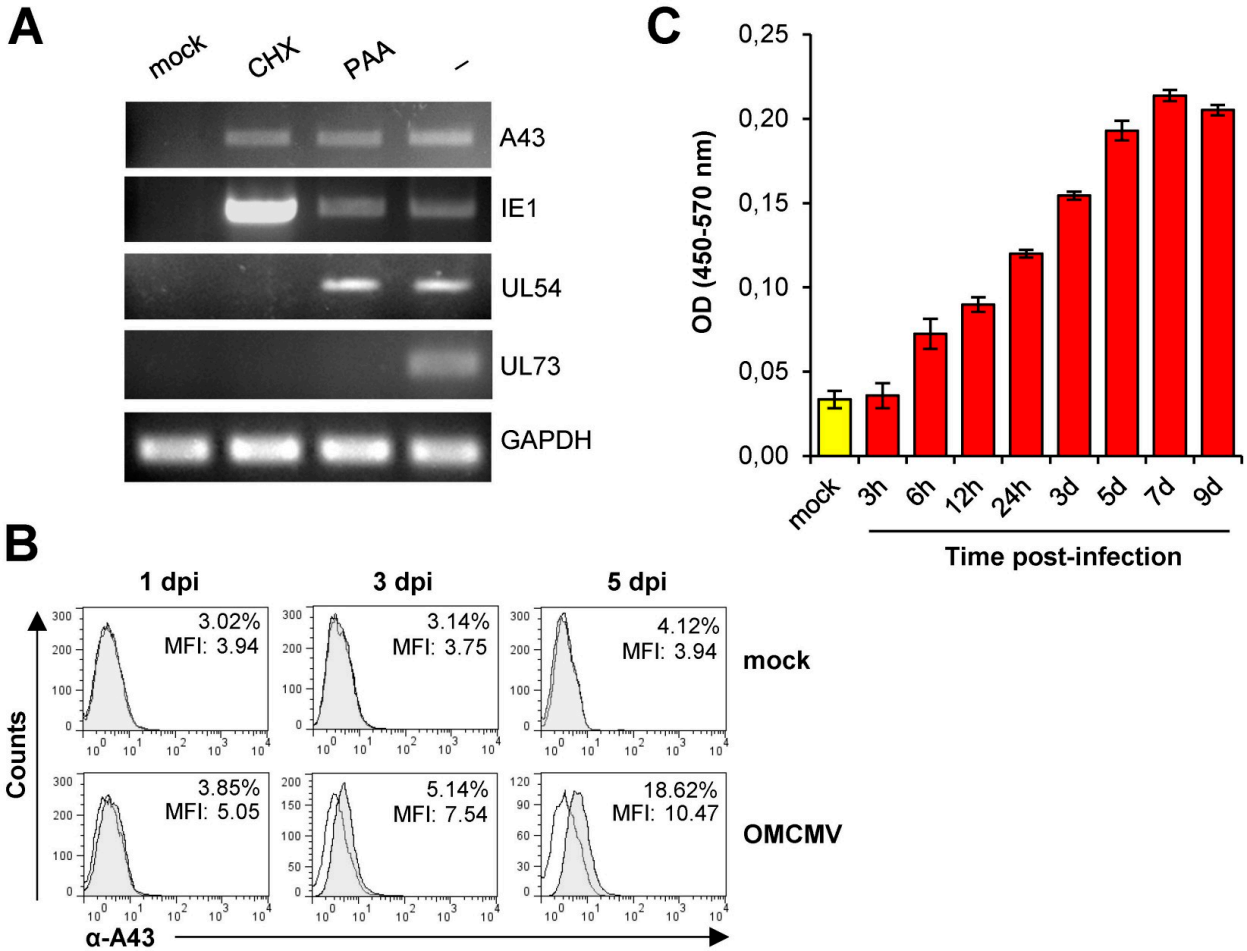
Expression of A43 in OMCV infected cells. (A) Expression of A43 mRNA during OMCMV infection. OMK cells were mock-infected or infected with OMCMV at an moi of 1 in the absence (-) or presence of CHX or PAA, as indicated. Whole-cell RNAs were reverse-transcribed, and PCRs were performed using primer sets specific for *A43*, *IE1*, *UL54*, *UL73* or *GAPDH*. Amplified products were separated on 1% agarose gels and visualized by RedSafe nucleic acid staining solution. (B) OMK cells mock infected or infected with OMCMV at an moi of 2 for 1, 3 or 5 days were analyzed by flow cytometry with the anti-A43 polyclonal antibody (shaded histograms) or an isotype control (open histograms). The percentage of positive cells and the MFI value for each sample are indicated. (C) Supernatants from OMK cells mock infected or infected with OMCMV at an moi of 2 for the different time points indicated were assessed by sandwich ELISA using the anti-A43 polyclonal antibody for soluble A43. Measurements (OD 450–570 nm) were performed in triplicate and a representative experiment of three is shown.

We first sought to analyze the expression of A43 in the context of the infection. To determine the kinetic class of this viral protein, owl monkey kidney (OMK) epithelial cells were mock-infected or infected with OMCMV at a moi of 1 in the absence or presence of two chemical inhibitors, cycloheximide (CHX) or phosphonoacetic acid (PAA), which prevent translation and viral DNA replication, respectively. Total RNA was extracted from the cultures and reverse transcriptase-mediated PCR (RT-PCR) analysis was performed using specific primers for this vCD48. As illustrated in Fig 1A, *A43* was detected under all conditions tested, including in the presence of CHX, when the immediate-early OMCMV gene *IE1* was abundantly expressed, but not the early *UL54* viral polymerase or the late *UL73* virion envelope glycoprotein N genes. Hence, *A43* can be considered an immediate early gene. We then examined the presence of the viral protein in infected cells. OMK cells, mock-infected or infected with OMCMV, were analyzed at different days after infection by flow cytometry using a polyclonal antibody that we raised against the viral protein. As shown in Fig 1B, at the three time points examined, 1, 3 and 5 days post infection, A43 could be detected at very low amounts at the surface of the infected cells. To directly investigate whether A43 was shed during infection, culture supernatants were collected at different times post-infection over a 9 day period and examined by ELISA using the A43 polyclonal antibody. Remarkably, and depicted in Fig 1C, A43 was found in the extracellular milieu since the first 6 h after infection and accumulated over time, thereby indicating that the A43 protein is released as a soluble molecule soon after infection.

### A43 is proteolytically processed through its transmembrane proximal region

**Fig 2:**
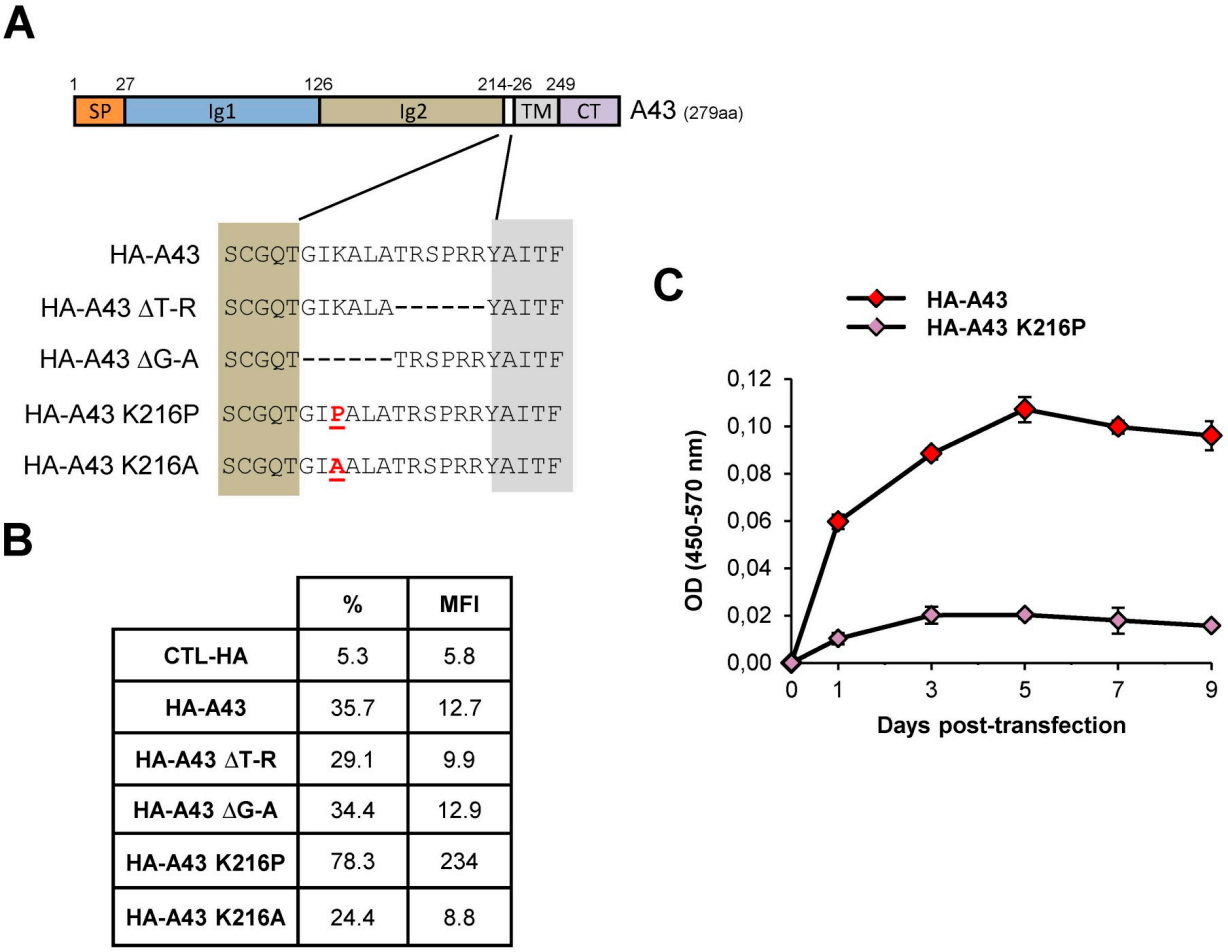
Shedding of A43. (A) Schematic representation of A43. SP, signal peptide; Ig1 and Ig2, immunoglobulin domains; TM, transmembrane domain; CT, cytoplasmic tail. The expanded region shows the amino acid sequence of the last five residues of Ig2 (brown shaded), the membrane proximal region and the first five residues of the TM region (gray shaded) of A43, in the HA-A43, HA-A43 ΔT-R, HA-A43 ΔG-A, HA-A43 K216P and HA-A43 K216A constructs, with the corresponding deleted (-) or mutated (in red and underlined) residues. (B) COS-7 cells transfected with the plasmids indicated in A or the corresponding empty vector (CTL-HA), were analyzed 24 h after transfection by flow cytometry, staining with an anti-HA mAb or an isotype control. The percentage of positive cells and the MFI value for each sample are indicated. (C) The supernatants of COS-7 cells transfected with HA-A43 or HA-A43 K216P were harvested at the indicated time points and assessed by sandwich ELISA for soluble A43 using the anti-A43 polyclonal antibody and an anti-HA mAb. Measurements (OD 450–570 nm) were performed in triplicate and a representative experiment is shown.

Ectodomain shedding of transmembrane proteins primarily occurs by proteolytic cleavage at sites in close proximity to the cell surface. In this process, the length of the proximal region and the structure of the cleavage site region of the protein tend to be more relevant than the specific amino acid sequence [24]. A43 contains a 12 aa-long membrane proximal (MP) region (Fig 2A). Therefore, to gain insight into the cleavage site of A43, and assess whether the integrity of the MP region was fundamental in this process, different mutants of the viral protein containing deletions or point mutations in this region were engineered. To this end, we used an HA-tagged A43 plasmid, encoding the viral protein with the hemagglutinin (HA) tag positioned immediately after its signal peptidase cleavage site. In two of the variants the length of the MP region was shortened, deleting either the six aa immediately juxtaposed to the transmembrane domain (aa 214–219, in plasmid HA-A43 ΔT-R), or the six aa more distal (aa 220–225, in plasmid HA-A43 ΔG-A) to this region (Fig 2A). In addition, the lysine at position 216 within the A43 MP region was substituted by a proline, which would be expected to disrupt its secondary structure, or alternatively by an alanine as a control, in mutant plasmids HA-A43 K216P and HA-A43 K216A, respectively (Fig 2A). COS-7 cells were transiently transfected with wild type HA-A43 or the constructed plasmids and the effects of the removed amino acid stretches or the mutated lysine residue were quantitatively assessed by measuring HA-A43 surface expression by flow cytometry. Surprisingly, there were no substantial differences in cell surface expression between the deletion mutants HA-A43 ΔT-R or HA-A43 ΔG-A and the wild type HA-A43 protein (Fig 2B). In contrast, a pronounced increase of surface staining was observed in cells transfected with the HA-A43 K216P mutant, but not with HA-A43 K216A. Consistent with these results, when we directly evaluated the release of soluble A43 by a sandwich ELISA of supernatants from COS-7 cells transfected with HA-A43 or HA-A43 K216P, at various time points, constitutive shedding of HA-A43, but not of HA-A43 K216P, was evident at every time point analyzed, from day 1 to 9 (Fig 2C). Altogether, these results indicate the absence of key particular MP sequences required for the proteolytic cleavage of A43, and suggest that mutation of lysine 216 to a proline may induce a conformational change resulting in impaired shedding of A43.

### A43 binds with high affinity and slow dissociation kinetics to host 2B4

**Fig 3:**
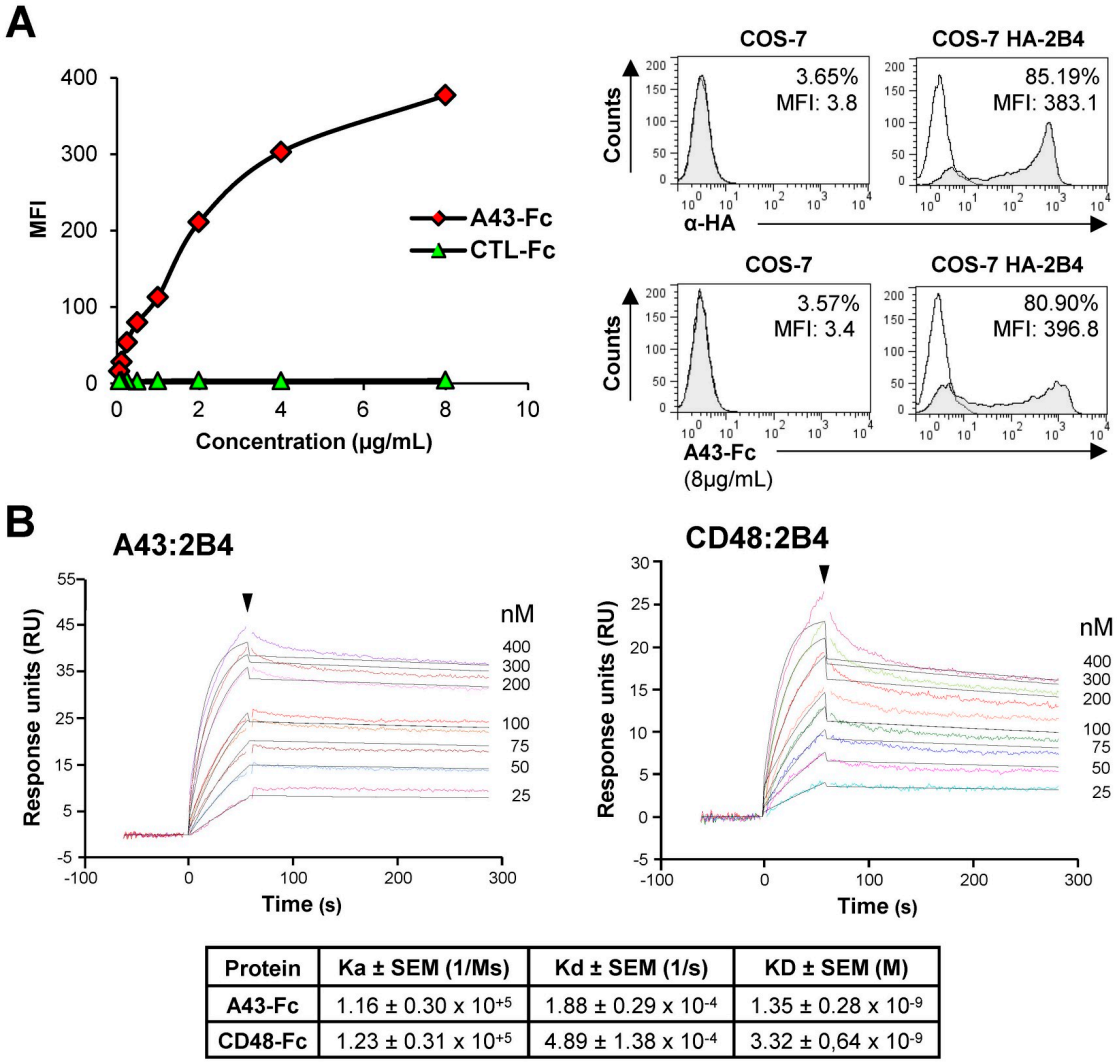
Soluble A43 efficiently interacts with host 2B4. (A) COS-7 cells nontransfected or transfected with host 2B4 (HA-2B4) were incubated with the different concentrations indicated of A43-Fc or an irrelevant Fc protein (CTL-Fc), and then analyzed by flow cytometry. Graph on the left: curves of MFI values for the interactions of the different concentrations of Fc fusion proteins with 2B4-transfected cells. Right top panel: cell staining with anti-HA mAb (shaded histograms) or isotype controls (open histograms). Right bottom panel: histograms corresponding to the data obtained using 8 μg/ml of A43-Fc (shaded histograms) or isotype controls (open histograms). The percentage of positive cells and MFI values are indicated in each histogram. (B) An example of the binding sensorgrams and fittings obtained for the determination of the kinetic constants of the A43:host 2B4 and host CD48:2B4 interactions by SPR analysis is shown. Binding and dissociation of several concentrations of analytes at 30 μl/min were recorded and adjusted to a 1:1 Langmuir fitting (solid lines). The nanomolar concentration corresponding to each sensorgram is indicated. The arrowhead points the end of the injection. The mean values ± SEM of the kinetic parameters and derived affinity constants of two independent experiments are indicated in the table below.

To study in more detail the interaction of A43 with 2B4, we generated an A43-Fc fusion protein, composed by the extracellular region of A43 fused to the Fc domain of the human IgG_1_. We first confirmed by flow cytometry the capacity of A43-Fc to bind host (*Aotus trivirgatus*) 2B4 by incubating increasing amounts of the fusion protein with COS-7 cells transiently transfected with host 2B4, using an expression vector encoding an N-terminal HA-tagged version of this receptor. As shown in Fig 3A (left graph), A43-Fc efficiently interacted, in a dose-dependent manner, with cell surface host 2B4. Indeed, the observed number of 2B4-transfected COS-7 cells binding A43-Fc (80.90%) was comparable to the percentage of COS-7 cells expressing 2B4 (85.19%) at the highest dose of A43-Fc evaluated (Fig 3A, compare upper and bottom panels in the right). Next, we carried out surface plasmon resonance (SPR) kinetic analyses to quantify in real time the biochemical interaction of A43 and host 2B4. To this end, host 2B4-Fc fusion protein was immobilized in the sensor chip and the capacity to bind different concentrations of soluble A43-Fc was examined. In addition, host CD48-Fc was employed as a control, allowing us to compare the interaction of A43:host 2B4 with that of host CD48:host 2B4, as illustrated in Fig 3B. The binding kinetics revealed that A43 interacts with host 2B4 with an association rate (K_a_) of 1.16 x 10^5^ M^-1^ s^-1^ and a dissociation rate (K_d_) of 1.88 x 10^−4^ s^-1^, yielding an equilibrium dissociation constant (K_D_) of 1.3 nM. These results evidenced a high affinity and stable interaction between A43 and host 2B4. When compared to control host CD48, the kinetic measurements were quite similar (K_D_ of 3.3 nM), with A43 binding host 2B4 with a 2.4-fold higher affinity, and dissociating 2.6-times slower.

### Host CD48:2B4 interaction is competed by A43

**Fig 4:**
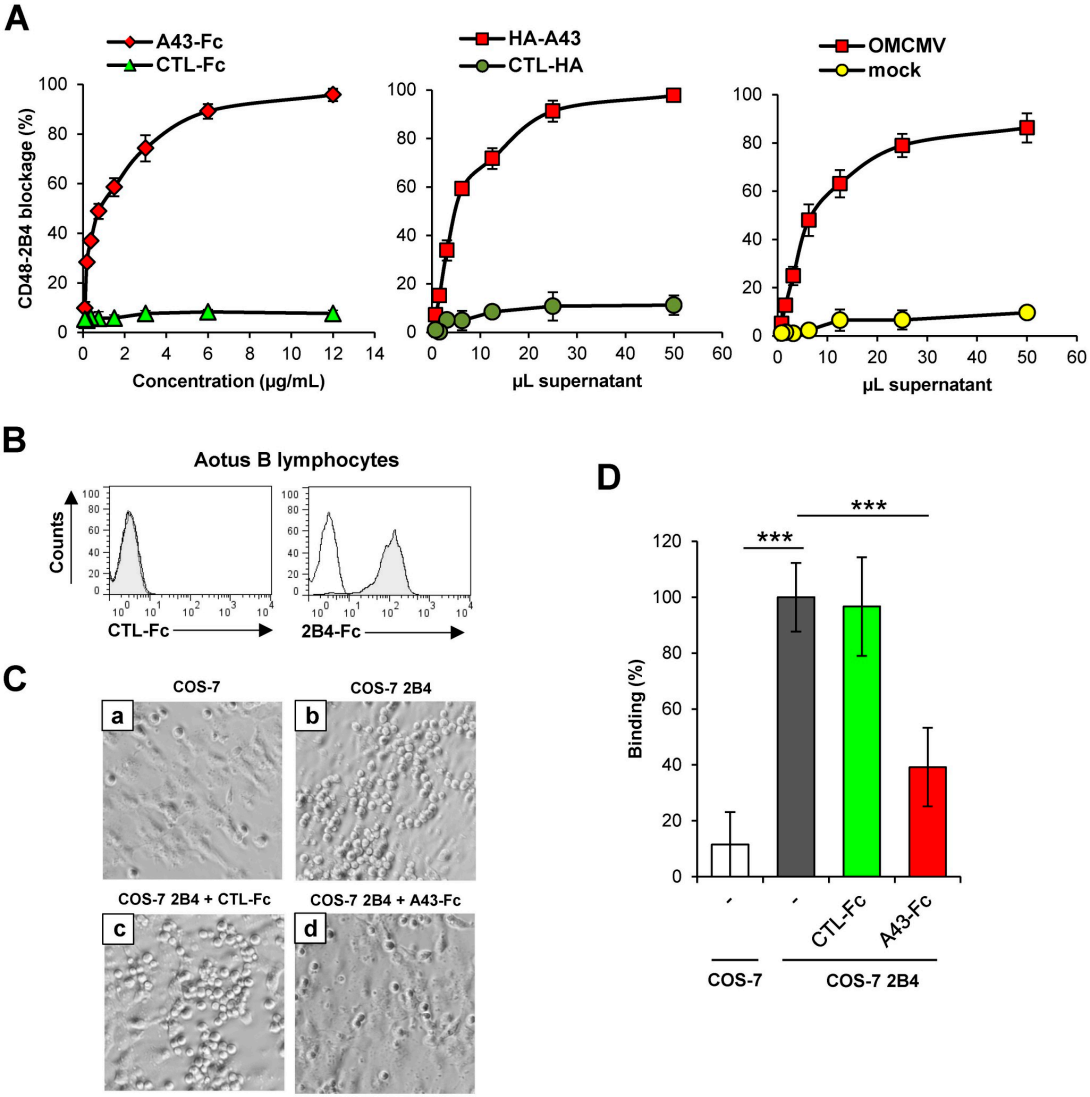
A43 blocks host 2B4:CD48 binding. (A) Plates coated with host 2B4-Fc were incubated with the indicated concentrations of A43-Fc or CTL-Fc (left graph), different volumes of concentrated supernatants from COS-7 cells transfected with HA-A43 or the corresponding empty plasmid (CTL-HA; middle graph), or different volumes of supernatants derived from OMK cells mock infected or infected with OMCMV at an moi of 2 for 6 days (right graph), followed by biotinylated host CD48-Fc, and analyzed by sandwich ELISA. Measurements (OD 450–570 nm) were performed in triplicate and the percentages of blockade of host 2B4-CD48 binding were calculated from the ODs obtained. (B) Flow cytometry analysis of *A*. *trivirgatus* B lymphocytes incubated with 8 μg/mL of host 2B4-Fc or an unrelated CTL-Fc fusion protein, and then analyzed by flow cytometry (shaded histograms). (C) COS-7 cells transfected with host 2B4 (panels b-d) or the corresponding empty plasmid (panel a) were incubated for 1 h with *A*. *trivirgatus* B lymphocytes at 4°C. When indicated, host B lymphocyes were pre-incubated for 30 min with 10 μg/mL of CTL-Fc protein (panel c) or A43-Fc (panel d). Cultures were examined under an inverted microscope and one representative experiment out of three is shown (20X magnification). (D) COS-7 cells transfected with HA-2B4 (COS-7 2B4) or the corresponding empty plasmid (COS-7) were incubated for 1 h at 4°C with host B lymphocytes previously labelled with calcein. When indicated, host B lymphocytes were pre-incubated for 30 min with 10 μg/mL of CTL-Fc or A43-Fc proteins. After the co-culture, cells were detached with trypsin, and analyzed by flow cytometry. *A*. *trivirgatus* B lymphocytes and COS-7 cells were gated based on forward and side scatter and examined for calcein staining. The percentage of binding of labeled host B lymphocytes in each sample relative to the untreated sample is represented. The assay was performed in sixplicate and the asterisks indicate that the difference between both points is statistically significant. *** p <0.001.

We next explored whether soluble A43 was able to block the host CD48:2B4 interaction. To directly prove this, we employed an ELISA assay, in which the host 2B4-Fc protein immobilized on the plate was exposed to increasing amounts of A43-Fc and subsequently incubated with biotinylated host CD48-Fc. As shown in Fig 4A (left graph), the A43-Fc protein markedly decreased the interaction of host CD48 to its countereceptor. Similar results were also obtained when the assays were performed using supernatants of COS-7 cells transfected with HA-A43 (Fig 4A, middle graph). Moreover, we assessed whether this viral protein produced during infection was also capable to prevent the interaction of host CD48 with host 2B4. Notably, we found that A43 containing supernatants from infected cells also resulted in an efficient and dose-dependent inhibition of the binding of biotinylated host CD48-Fc to the host 2B4-Fc protein (Fig 4A, right graph).

We further studied the ability of the A43-Fc fusion protein to compete the host 2B4:CD48 interaction when both receptors were expressed at the cell surface, by carrying out cell binding assays. We employed host EBV-transformed B lymphocytes, which express CD48 (Fig 4B), and COS-7 cells transiently transfected with host 2B4. The binding of these B lymphocytes to host 2B4-expressing COS-7 cells or untransfected control COS-7 cells was assessed in the presence or absence of A43, by analyzing a number of fields under the light microscope. B lymphocytes showed a specific binding to host 2B4-expressing COS-7 cells (Fig 4C, compare panels a and b). Importantly, when the host 2B4^+^ COS-7 cells were incubated with A43-Fc, a substantial decrease of the interaction between these cells and B lymphocytes was observed (Fig 4C, panel d), whereas this interaction was unaltered after treatment with an unrelated Fc protein (CTL-Fc; Fig 4C, panel c). To quantitatively measure these effects, cell binding assays were performed using host B lymphocytes previously labelled with calcein, allowing the determination of the number of bound B cells by flow cytometry. As observed before, while the binding of B cells to COS-7 cells transfected with host 2B4 was not significantly affected by the presence of the control fusion protein, it markedly decreased after addition of A43-Fc, yielding an inhibition of around 60% of the host 2B4-induced binding (Fig 4D). Taken together, these findings demonstrate that soluble purified A43 can impair the interaction between cells expressing host 2B4 and host CD48^+^ cells.

### A43 efficiently binds to human 2B4

**Fig 5:**
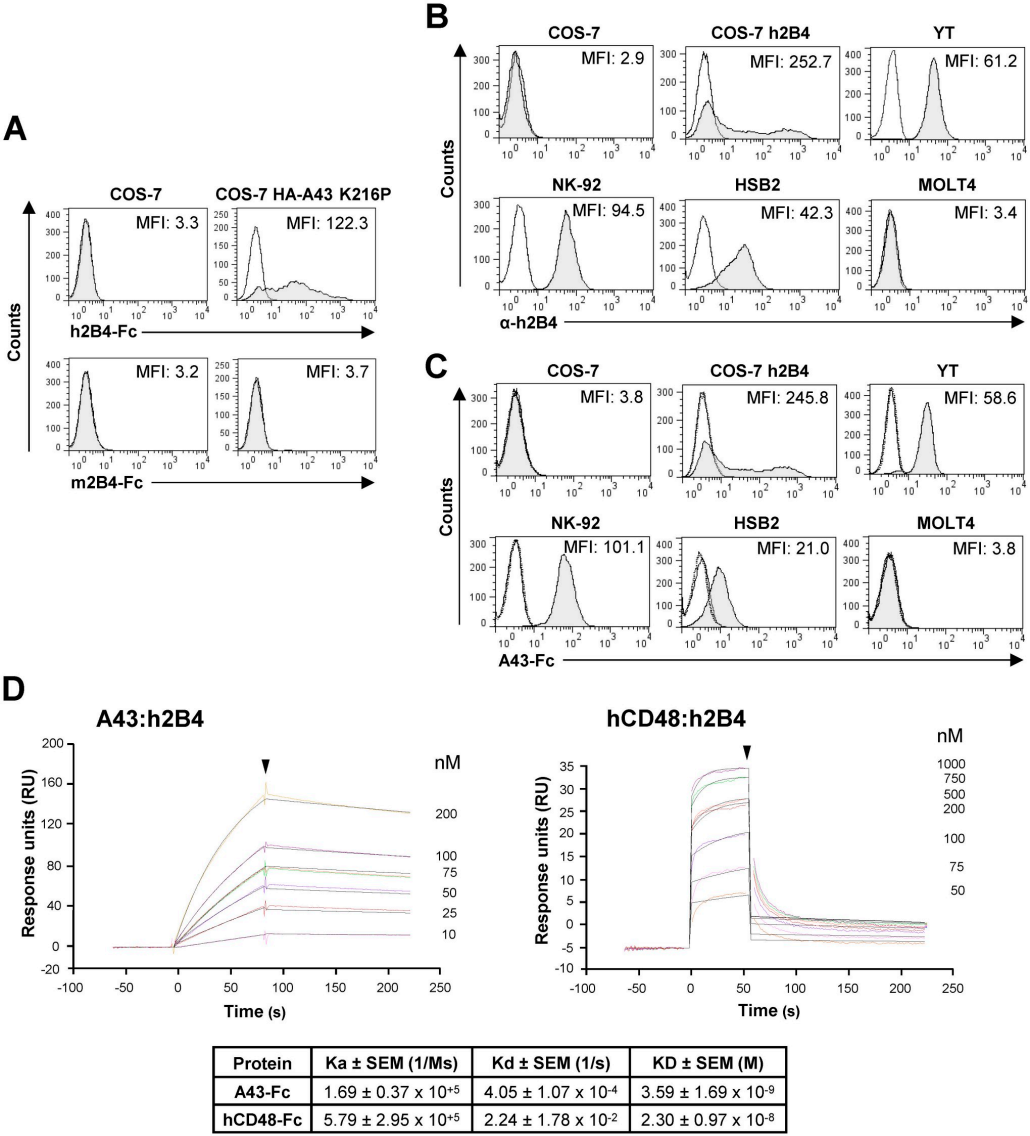
Analysis of the binding of A43 to h2B4. (A) Flow cytometry analysis of COS-7 cells nontransfected or transfected with HA-A43 K216P and incubated with 8 μg/mL of human 2B4-Fc (h2B4-Fc) or murine 2B4-Fc (m2B4-Fc) fusion proteins, and then analyzed by flow cytometry (shaded histograms). (B) Flow cytometry analysis of COS-7 cells nontransfected or transfected with h2B4, or the cell lines YT, NK-92, HSB2, or MOLT4, stained with anti-h2B4 mAb (α-h2B4, shaded histograms). (C) Cells indicated in B were incubated with 8 μg/mL of A43-Fc (shaded histograms) or an irrelevant CTL-Fc protein (dotted line open histograms) and then analyzed by flow cytometry. In A, B and C, solid line open histograms represent isotype controls. MFIs are indicated in each histogram. (D) An example of the binding sensorgrams and fittings obtained for the determination of the kinetic constants of the A43:h2B4 and hCD48:h2B4 interactions by SPR analysis is shown. Binding and dissociation of several concentrations of analytes at 30 μl/min were recorded and adjusted to a 1:1 Langmuir fitting (solid lines). The nanomolar concentration corresponding to each sensorgram is indicated. The arrowhead points the end of the injection. The mean values ± SEM of the kinetic parameters and derived affinity constants from three (A43:h2B4 interaction) or four (hCD48:h2B4 interaction) independent experiments are indicated in the table below.

We then decided to investigate whether A43 displayed cross-species receptor binding properties, by analyzing its interaction with human and mouse 2B4 (h2B4 and m2B4, respectively). To this end, COS-7 cells transiently transfected with the plasmid HA-A43 K216P, which allows high expression levels of A43 at the cell surface, were tested by flow cytometry in binding assays performed with the h2B4-Fc or m2B4-Fc fusion proteins. Fig 5A shows that while m2B4-Fc did not interact with A43, a strong binding activity of h2B4-Fc to the viral protein was observed. Human 2B4 is mainly expressed in NK cells, although it is also present at lower levels on other cytotoxic cells, such as CD8^+^ T cells, γδ T cells, basophils, and eosinophils. Taking this in consideration, we evaluated the capacity of A43-Fc to bind different human cell lines constitutively expressing h2B4, either derived from NK cells (NK-92 and YT) or T lymphocytes (HSB2) (Fig 5B). In these assays, COS-7 cells transiently transfected with h2B4 were used as a positive control, whereas the human T lymphoblast cell line MOLT-4 that does not express h2B4 and untransfected COS-7 cells were employed as negative controls. As illustrated in Fig 5C, A43 displayed a clear reactivity with the four cell lines tested that expressed 2B4 (NK-92, YT, HSB2, and h2B4-transfected COS-7 cells), staining them at nearly the same extension than the h2B4 specific monoclonal antibody (mAb; compare MFI values in corresponding panels in Fig 5B and 5C). In contrast, no interaction of the viral protein was detected for the human cell line MOLT-4 or the untransfected COS-7 cells.

We also carried out SPR kinetic analyses to quantify in real time the A43:h2B4 interaction in a similar way as described for the interaction of A43 with host 2B4, but in this case immobilizing h2B4-Fc in the sensor chip. Thus, we assessed the ability of h2B4 to bind different concentrations of soluble A43-Fc, using hCD48-Fc as a control. Fig 5D, shows an example of the sensorgrams fittings generated to obtain the affinity constants. The binding kinetics indicated that A43 binds to h2B4 with an association rate (K_a_) of 1.69 x 10^5^ M^-1^ s^-1^ and a dissociation rate (K_d_) of 4.05 x 10^−4^ s^-1^, resulting in an equilibrium dissociation constant (K_D_) of 3.6 nM. As we previously found for the binding of A43 to host 2B4, these results evidenced an interaction of high affinity and stability between A43 and h2B4. Remarkably however, and in contrast to the findings obtained in the host context, A43 was found to bind h2B4 with a 6-fold higher affinity and dissociate 55-fold slower than hCD48.

### Soluble A43 prevents h2B4-mediated NK-cell adhesion to target cells

**Fig 6:**
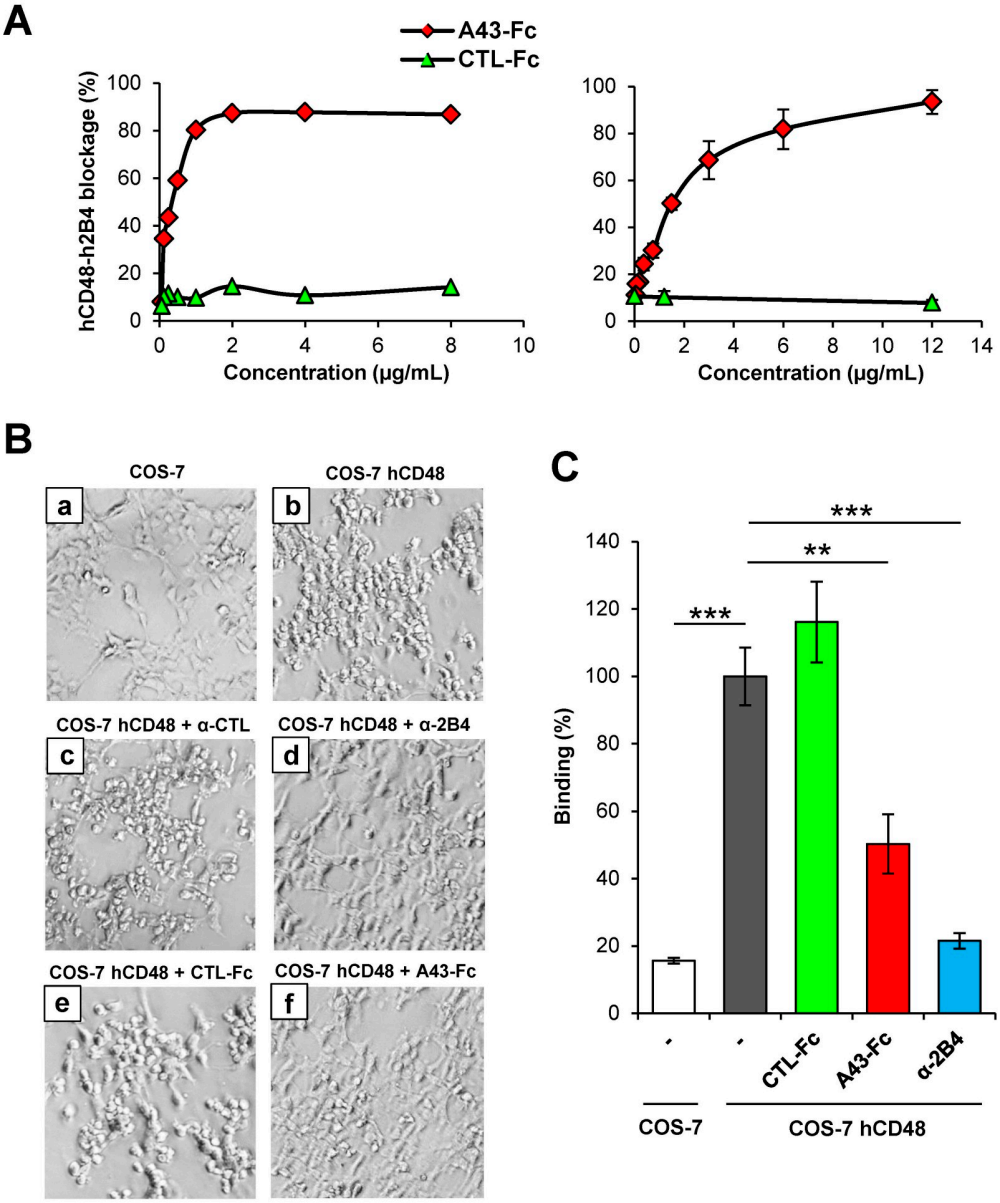
A43 is able to block h2B4-mediated NK cell adhesion. (A) Left graph: Flow cytometry analysis of NK-92 cells incubated with the indicated concentrations of A43-Fc or an irrelevant CTL-Fc protein, followed by biotinylated hCD48-Fc. Right graph: Plates coated with h2B4-Fc were incubated with the indicated concentrations of A43-Fc or CTL-Fc, followed by biotinylated hCD48-Fc, and analyzed by sandwich ELISA. One representative experiment of each assay is shown. The percentages of blocking interaction were calculated from the percentages of binding measured by flow cytometry or the ODs obtained by ELISA in the absence of A43-Fc or CTL-Fc proteins. (B) COS-7 cells transfected with hCD48 (panels b-f) or the corresponding empty plasmid (panel a) were incubated for 1 h with NK-92 cells at 4°C. When indicated, NK-92 cells were pre-incubated for 30 min with 10 μg/mL of an unrelated control mAb (α-CTL, panel c), α-h2B4 mAb (panel d), CTL-Fc protein (panel e), or A43-Fc (panel f). Cultures were examined under an inverted microscope and one representative experiment out of three is shown (20X magnification). (C) Same as in part B, except that NK-92 cells were stained with calcein prior to incubation with the antibody or the fusion proteins and, after the co-culture, cells were detached with trypsin, and analyzed by flow cytometry. NK-92 cells and COS-7 cells were gated based on forward and side scatter and examined for calcein staining. The percentage of binding of labeled NK-92 cells in each sample relative to the untreated sample is represented and the standard deviations of three independent samples are shown. The asterisks indicate that the difference between both points is statistically significant. ** p <0.01, *** p <0.001.

The observation that A43 interacts with human 2B4 does not only represent an important finding by itself, but in addition enabled us to pursue functional studies using 2B4^+^ human NK cells. Thus, in the next set of experiments we sought to determine whether soluble A43 was a functionally active molecule. Since 2B4 is an important co-activating receptor for NK cell function, we analyzed the capacity of A43 to interfere with NK cell activities. First, we confirmed the potential of A43-Fc to block the hCD48:h2B4 interaction, performing cytofluorimetric assays using the human NK-92 cell line and biotinylated hCD48-Fc. As shown in Fig 6A (left graph), A43-Fc was able to efficiently compete in a dose-dependent manner the binding of hCD48 to h2B4. Comparable results were obtained by sandwich ELISA, when the ability of different doses of A43-Fc to block the binding between the h2B4-Fc protein (that coated the plates) and biotinylated hCD48-Fc was assessed (Fig 6A, right graph).

Target cell adhesion is an indispensable event for NK cell activation, and the 2B4:CD48 interaction has been shown to contribute to this process [25]. To evaluate the effects of A43 on NK cell adhesion, we performed cell binding assays using COS-7 cells transiently transfected with hCD48 and NK-92 cells. The binding of NK-92 cells to hCD48-expressing COS-7 cells or untransfected control COS-7 cells was determined in the presence or absence of A43, by examining a number of fields under the microscope. As expected, NK-92 cells exhibited a specific binding to hCD48-expressing COS-7 cells (Fig 6B, compare panels a and b). Rosettes of NK cells adhering to the cell surface of the COS-7 cells transfected with hCD48 could be observed. The specificity of this CD48-induced adhesion was further confirmed using a blocking anti-h2B4 mAb. In this case, binding abrogation to almost background levels (observed in COS-7 cells and shown in Fig 6B, panel a) was found upon preincubation of the NK-92 cells with the anti-h2B4 mAb, but not with an isotype control mAb (see panels c and d in Fig 6B). Notably, when NK-92 cells were exposed to A43-Fc, a marked reduced interaction between these cells and hCD48-transfected COS-7 cells was observed, with barely any visible rosette of NK-92 cells in the cultures (Fig 6B, panel f). In contrast, cell adhesion remained unaffected after incubation with an unrelated Fc protein (CTL-Fc; Fig 6B, panel e). We then proceeded to quantitatively measure these effects by flow cytometry using NK-92 cells labelled with calcein. As observed before, while the binding of NK-92 cells to COS-7 cells transfected with hCD48 was not altered significantly after incubating the NK-92 cells with an unrelated fusion protein, it substantially decreased after addition of A43-Fc, resulting in an inhibition of around 50% of the hCD48-induced binding (Fig 6C). Again, the specificity of the binding, abrogating nearly 80% of the hCD48-induced adhesion, was proven with the mAb directed to h2B4. These data demonstrate that soluble A43 can function interfering with h2B4-mediated NK cell adhesion to target cells.

### Soluble A43 blocks conjugate and the immune synapse formation between human NK cells and hCD48-expressing target cells

**Fig 7:**
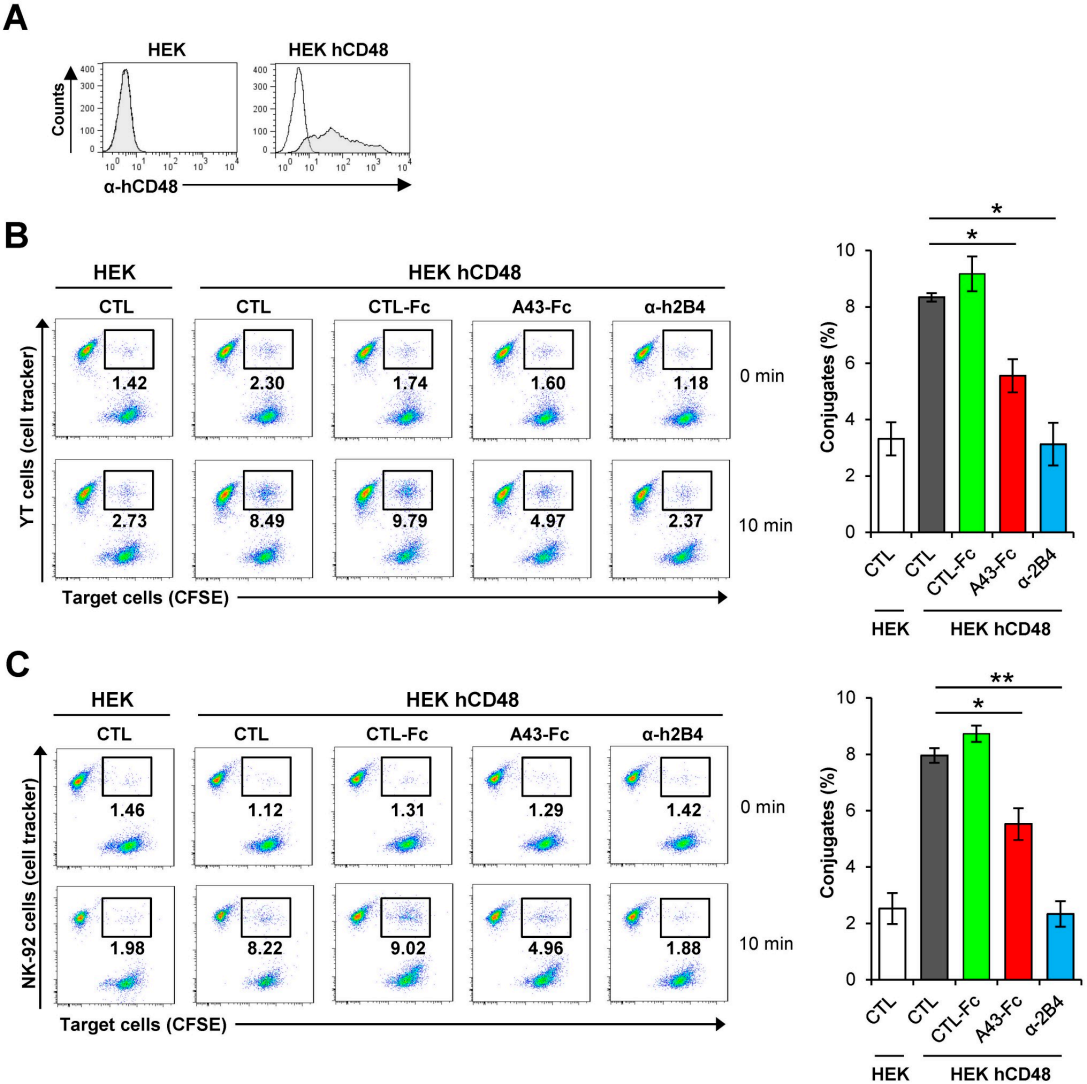
A43 impairs conjugate formation. (A) Flow cytometry analysis of HEK cells transfected with hCD48 (HEK hCD48) or the corresponding empty plasmid (HEK) and stained with anti-hCD48 mAb (shaded histograms) or an isotype control (open histograms). (B) CFSE-labeled HEK cells transfected with hCD48 or empty plasmid were incubated at 37°C with YT cells stained with CellTracker Blue, and the formation of conjugates analyzed after 0 or 10 min. When indicated, YT cells were pre-incubated for 30 min with 10 μg/mL of an irrelevant CTL-Fc protein, A43-Fc, or α-2B4 mAb, or left untreated (CTL). Conjugates were identified as double positive cells by flow cytometry. Dot plots of a representative experiment are depicted in the left panels (with the percentage of conjugates indicated in each case), and the right panel shows a graphic representation of the conjugates formed at 10 min of two independent assays. (C) Same as in part B, except that NK-92 cells were used instead of YT cells. The asterisks indicate that the difference between both points is statistically significant. * p <0.05, ** p <0.01.

Formation of conjugates between NK and target cells is an important step required for efficient NK cell activation and subsequent cytolytic functions. We therefore examined the impact of the binding of soluble A43 to NK cells on conjugate formation. To this end, we transfected HEK cells with hCD48 (see Fig 7A) and employed a flow cytometry-based assay that enabled to determine the frequency of the stable conjugates formed between these target cells and YT cells after being labelled with two distinct fluorophores (cell tracker for YT cells and CFSE for target cells). Conjugates were identified after co-incubating for 10 min these labelled cells in suspension as double fluorescent events. As illustrated in Fig 7B, after this incubation time, the expression of CD48 on the surface of HEK cells conferred to these cells the ability to form conjugates with YT cells. Notably, exposure of the YT cells to A43-Fc, but not of an unrelated fusion protein, provoked a pronounced reduction of conjugate formation with hCD48-expressing HEK cells. As a control, the anti-h2B4 blocking mAb was also included in the assay and shown to drastically diminish the proportion of conjugates to levels comparable to those observed after incubation with HEK cells (Fig 7B). Identical results were observed when the assays were conducted with NK-92 cells instead of YT cells, further confirming that the binding of A43 to h2B4 in NK cells severely affects their ability to form conjugates with target cells (Fig 7C).

**Fig 8:**
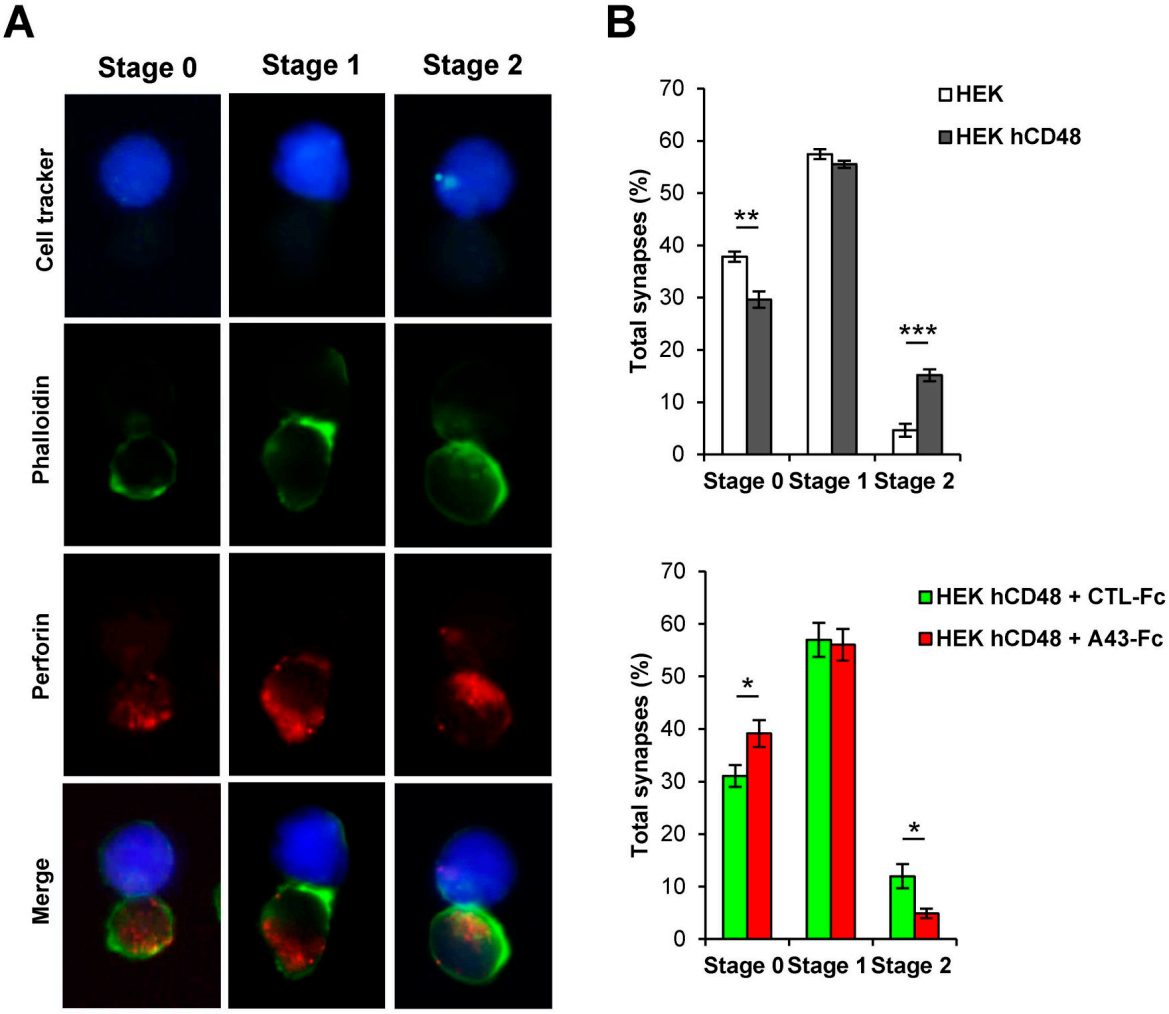
A43 interferes with the formation of the mature human NK cell immune synapse. HEK cells transfected with hCD48 (HEK hCD48) or the corresponding empty plasmid (HEK) and labelled with CellTracker Blue were incubated with YT cells at a 1:1 ratio for 10 min to allow conjugate formation. Cells were fixed and stained with phalloidin (to detect actin) and an anti-perforin mAb. Conjugates were then analyzed by microscopy, magnification 40X. (A) Individual stainings for a representative conjugate of each stage are shown. (B) Top panel: The percentages of YT cells at each stage of the formation of the immune synapse were determined and represented. At least 50 conjugates were analyzed for each condition, and the means and standard deviations of three independent samples are shown. Bottom panel: Same as in top panel, except that YT cells were pre-incubated with 10 μg/mL of A43-Fc or an irrelevant CTL-Fc protein for 30 min prior to incubation with HEK hCD48 target cells. Asterisks indicate statistically significant differences between samples. * p <0.05, ** p <0.01, *** p <0.001.

Following the interaction of the NK cell with the target cell, the establishment of a highly stable immunological synapse is needed to ensure the directed release of perforin-granzyme granules toward the target cell. 2B4 has been shown to rapidly accumulate at the cell-cell contact site and participate in the formation of the lytic NK cell immune synapse [26]. Thus, we next assessed the potential of A43 to interfere with the establishment of the mature cytotoxic NK cell immune synapse. To investigate this, we performed immunofluorescence microscopy assays on conjugates established between YT cells and target HEK cells transfected with hCD48 or the corresponding empty plasmid and labelled with the cell tracker, using phalloidin and an anti-perforin mAb to visualize NK polymerized actin and cytotoxic granules, respectively. We examined the occurrence of three different stages in the progression of the formation of the functional NK cell lytic immune synapse (Fig 8A). Stage 0, corresponding to conjugates in which actin and cytotoxic granules were not polarized toward the synapse. Stage 1, corresponding to conjugates in which polymerized actin, but not cytotoxic granules, accumulated at the synapse. And stage 2, corresponding to conjugates in which both polymerized actin and cytotoxic granules had reached the synapse. In this latter case, co-localization of phalloidin (actin) and perforin could be clearly visualized (right bottom panel in Fig 8A). We first evaluated the ability of the YT cells to form an immune synapse with hCD48-transfected HEK cells as compared to control HEK cells, analyzing the same number of conjugates. Fig 8B (upper panel) shows an augmented proportion of conjugates exhibiting fully polarized granules (stage 2) upon expression of hCD48 on the surface of the target cells. This effect was associated with a concomitant decrease of conjugates in stage 0, lacking acting polymerization and granule polarization. These data are consistent with a role of hCD48 promoting the progression of the NK immune synapse. Remarkably, when YT cells were pre-treated with the A43-Fc protein, a clear decrease of conjugates with hCD48-transfected HEK cells in stage 2 was found as compared to those established by YT cells pre-incubated with an unrelated Fc fusion protein (Fig 8B, bottom panel). In conclusion, A43 inhibits the formation of the mature cytotoxic NK cell immune synapse.

### Impairment of 2B4-dependent human NK cell cytotoxicity and IFN-γ production by soluble A43

**Fig 9:**
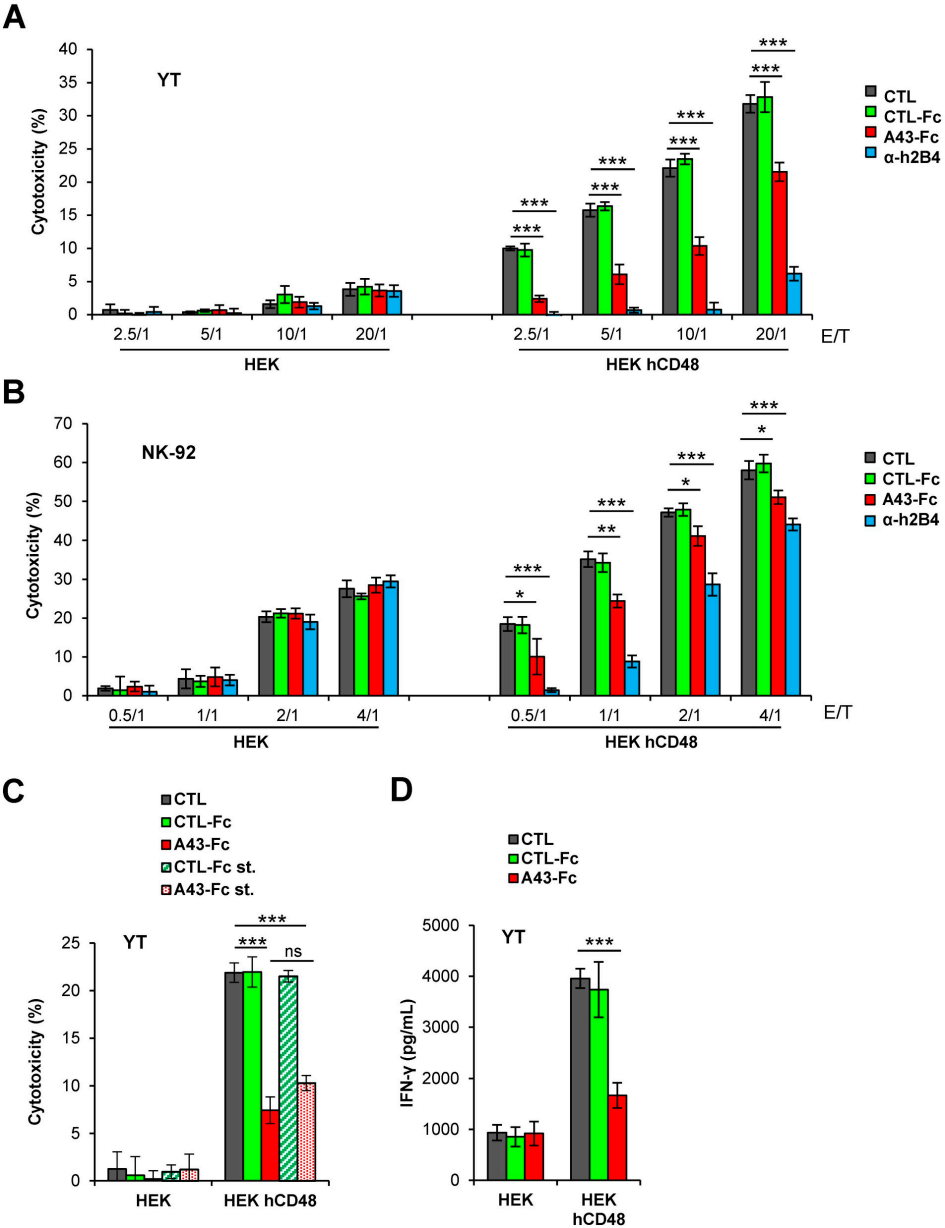
A43 inhibits YT and NK-92 cell cytotoxicity. (A) Calcein-labelled HEK cells transfected with hCD48 (HEK hCD48) or control empty plasmid (HEK) were incubated at 37°C with YT cells at different E/T ratios. When indicated, YT cells were pre-incubated for 30 min with 10 μg/mL of an irrelevant CTL-Fc protein, A43-Fc, α-h2B4 mAb or left untreated (CTL). Four hours later, the fluorescence emitted by the calcein released to the supernatant was measured and the percentage of cytotoxicity of the YT cells against the target cells calculated. The means and standard deviations of three independent samples are represented for each condition. (B) Same as in A, except that NK-92 cells were used instead of YT cells. (C) Same as in A at a 10/1 E/T ratio, except that, when indicated, YT cells were not pre-incubated with the fusion proteins but instead, A43-Fc or the CTL-Fc protein were added to the co-culture at the same time than the YT cells (simultaneous treatment; st) (D) Determination of the IFN-γ levels from the supernatants of the indicated co-cultures shown in C. Measurements were performed in triplicate. Asterisks indicate that the difference between both points is statistically significant, * p <0.05, ** p <0.01, *** p <0.001; and ns indicates non-significant.

We then investigated whether soluble A43 had functional consequences on NK cell-mediated lysis of target cells. We performed cytotoxicity assays using as effectors the YT cell line at various E/T ratios, and untransfected or hCD48-transfected HEK cells stained with calcein, as targets. As seen in Fig 9A, in these assays the killing displayed by the YT cells toward untransfected HEK cells was minimal. In contrast, the expression of hCD48 substantially stimulated killing of HEK cells by the YT cells at all the different ratios tested (Fig 9A). We then assessed the effects of pre-treating YT cells with the A43 fusion protein or the anti-h2B4 mAb as a control. As expected, a drastic reduction of the cytotoxicity was observed when the NK cell receptor was masked with the anti-h2B4 mAb, confirming that NK cell-mediated lysis of hCD48-transfected HEK cells is h2B4 dependent. Remarkably, preincubation of the YT cells with the A43-Fc protein, but not with an unrelated Fc fusion protein, significantly inhibited NK-cell-mediated lysis at every E/T ratio tested in 3 independent experiments (Fig 9A). Similar results were obtained when employing the NK-92 cell line as effector cells, although in this case the base-line killing of these cells toward untransfected HEK cells was higher, slightly reducing the window in which to visualize the contribution of the h2B4-NK dependent lysis (Fig 9B). Of note, the blockade exerted by the viral protein did not require the pre-treatment of the NK cells with the A43-Fc protein, since when the viral protein was simultaneously added with the hCD48-expressing target cells to the effector cells, the h2B4-dependent NK cytotoxicity was inhibited at the same levels (Fig 9C). To assess if reduced NK cytolytic functions by A43 were also associated with diminished NK cytokine production, we examined the ability of the YT cells to secrete IFN-γ during the 4h cytotoxicity assay. As shown in Fig 9D, the expression of hCD48 in HEK cells significantly enhanced IFN-γ secretion by YT cells as compared with the untransfected HEK cells. Furthermore, A43-Fc, but not the CTL-Fc protein, caused a profound decrease of the CD48-stimulated YT cell production of IFN-γ.

**Fig 10:**
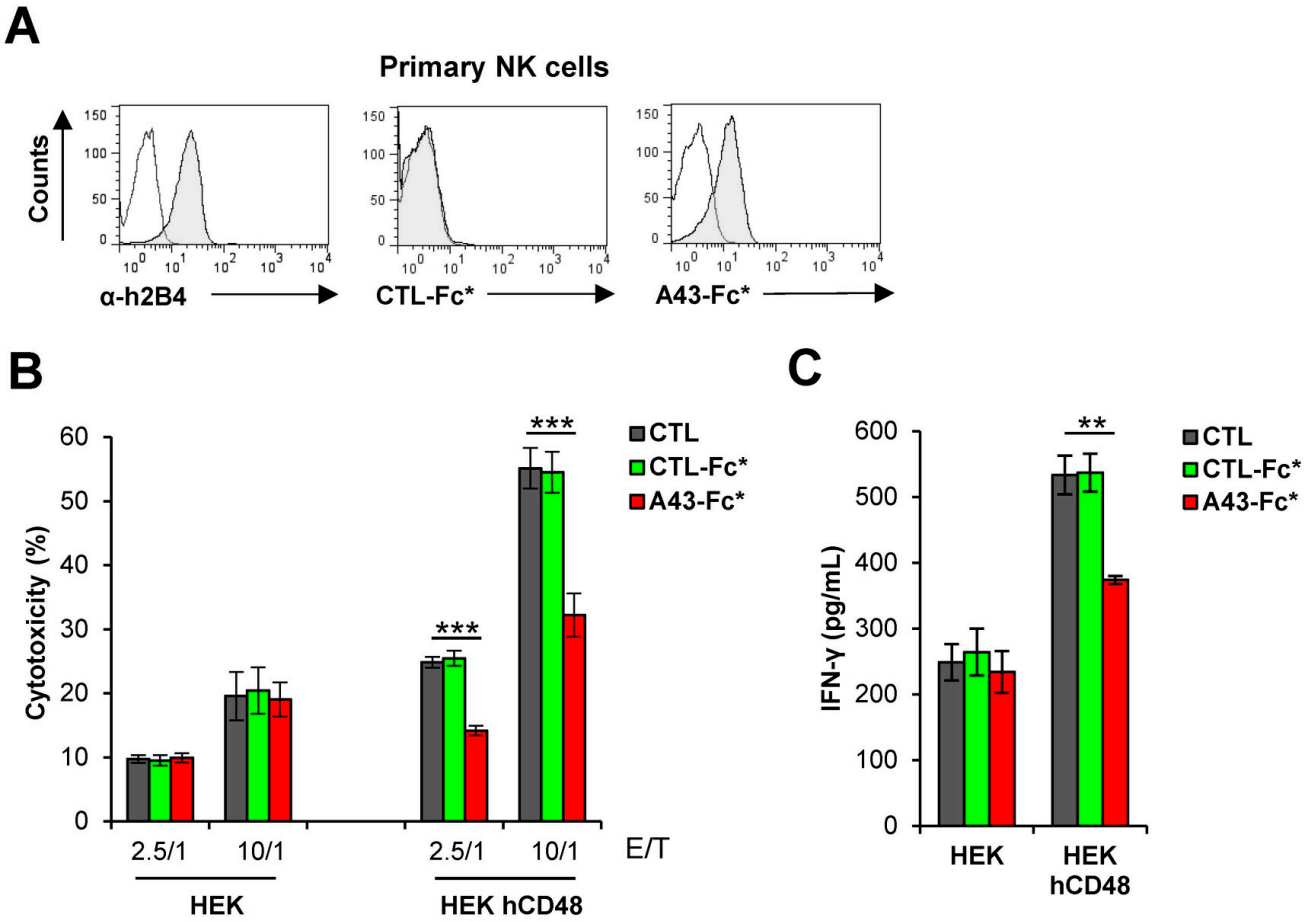
A43 limits the cytotoxic activity of primary NK cells. (A) Flow cytometry analysis of primary NK cells incubated with anti-2B4 mAb (α-h2B4), 8 μg/mL of an unrelated CTL-Fc* protein or A43-Fc*, and then analyzed by flow cytometry (shaded histograms). (B) Calcein-labelled HEK cells transfected with hCD48 (HEK hCD48) or control empty plasmid (HEK) were incubated at 37°C with primary NK cells at different E/T ratios. When indicated, NK cells were pre-incubated for 30 min with 10 μg/mL of an irrelevant CTL-Fc* protein, A43-Fc*, or left untreated. The percentage of cytotoxicity of the NK cells against the target cells was obtained as in Fig 9A. The means and standard deviations of three independent samples are represented for each condition. (C) Determination of the IFN-γ levels from the supernatants of the indicated co-cultures shown in B. Measurements were performed in triplicate. The asterisks indicate that the difference between both points is statistically significant, ** p <0.01, *** p <0.001.

We also examined the ability of soluble A43 to functionally block primary human NK cells. To this end, and to avoid potential complications derived from the use of human Fc fusion proteins and the Fc receptors present on the surface of the primary NK cells, we constructed a new version of the A43-Fc fusion protein, named A43-Fc*, containing six specific residues of the Fc region mutated that abrogate its binding to Fc receptors [27]. When examined by flow cytometry, A43-Fc*, but not an unrelated control Fc* fusion protein (CTL-Fc*), was capable to interact with the isolated primary blood NK cells (Fig 10A). As illustrated in Fig 10B, hCD48-transfected HEK cells were more susceptible to lysis by primary NK cells than untransfected HEK cells. Moreover, as it occurred in YT and NK-92 cells, a pronounced inhibition of the cytotoxicity was observed when the primary NK cells were preincubated with the A43-Fc* protein, but not with the CTL-Fc* protein, at the two different E/T ratio tested. In addition, treatment with A43-Fc* resulted in a significant and specific reduction of the hCD48-dependent NK cell production of IFN-γ (Fig 10C).

**Fig 11:**
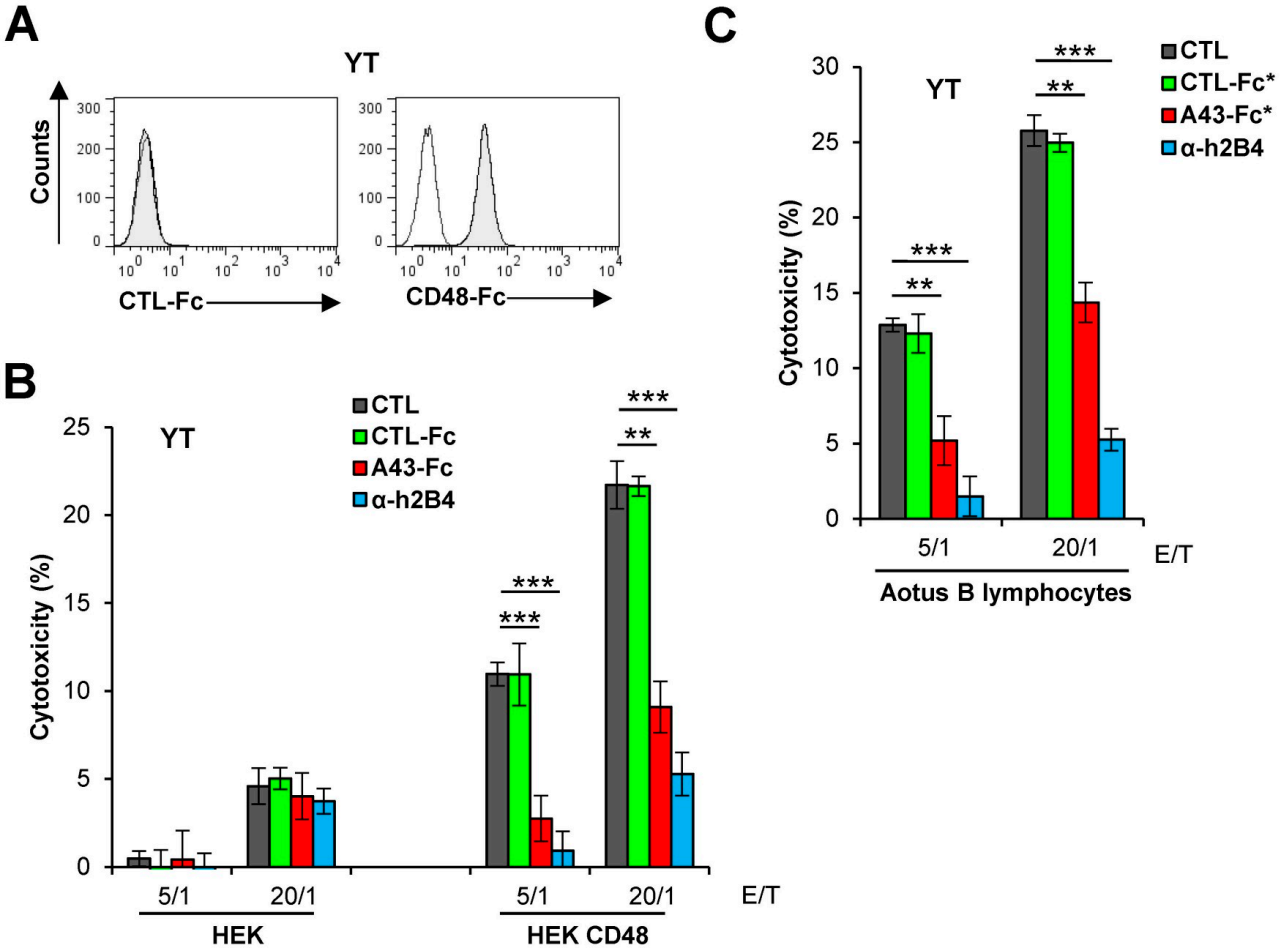
A43 impairs NK cell cytotoxicity mediated by host CD48. (A) Flow cytometry analysis of YT cells incubated with 8 μg/mL of an unrelated CTL-Fc protein or host CD48-Fc, and then analyzed by flow cytometry (shaded histograms). (B) Calcein-labelled HEK cells transfected with HA-CD48-Tm (HEK CD48) or control empty plasmid (HEK) were incubated at 37°C with YT cells at different E/T ratios. When indicated, NK cells were pre-incubated for 30 min with 10 μg/mL of an irrelevant CTL-Fc protein, A43-Fc, α-2B4 mAb, or left untreated. The percentage of cytotoxicity of the NK cells against the target cells was obtained as in Fig 9A. (C) Same as in B, except that target cells were host B lymphocytes, and CTL-Fc* or A43-Fc* proteins were used. The means and standard deviations of three independent samples are represented for each condition. The asterisks indicate that the difference between both points is statistically significant. ** p <0.01, *** p <0.001.

Due to the impossibility of obtaining host NK cells, and therefore in the absence of a cell system that allowed us to assess the activity of A43 on host NK cell functions, we analyzed if A43 was able to inhibit human NK cell cytotoxicity towards target cells expressing host CD48. For this purpose, we first confirmed by flow cytometry that YT cells could bind host CD48 (Fig 11A). Thereafter, we performed cytotoxicity assays employing as targets either HEK cells transfected with host CD48 or the immortalized host B lymphocytes. As shown in Fig 11B, YT cell lysis was higher in host CD48-expressing HEK cells than in HEK cells. Importantly, a substantial and specific block of the h2B4-dependent NK cell killing was observed when the YT cells were treated with A43-Fc at the two E/T ratios analyzed. Moreover, comparable results were observed when the host B lymphocytes were used as targets (Fig 11C). Thus, altogether the results indicate that soluble A43 may act protecting CD48-expressing cells from 2B4-dependent NK cell production of IFN-γ and cytotoxicity.

### The A43 protein secreted during viral infection reduces human NK cell killing against hCD48-expressing target cells

**Fig 12:**
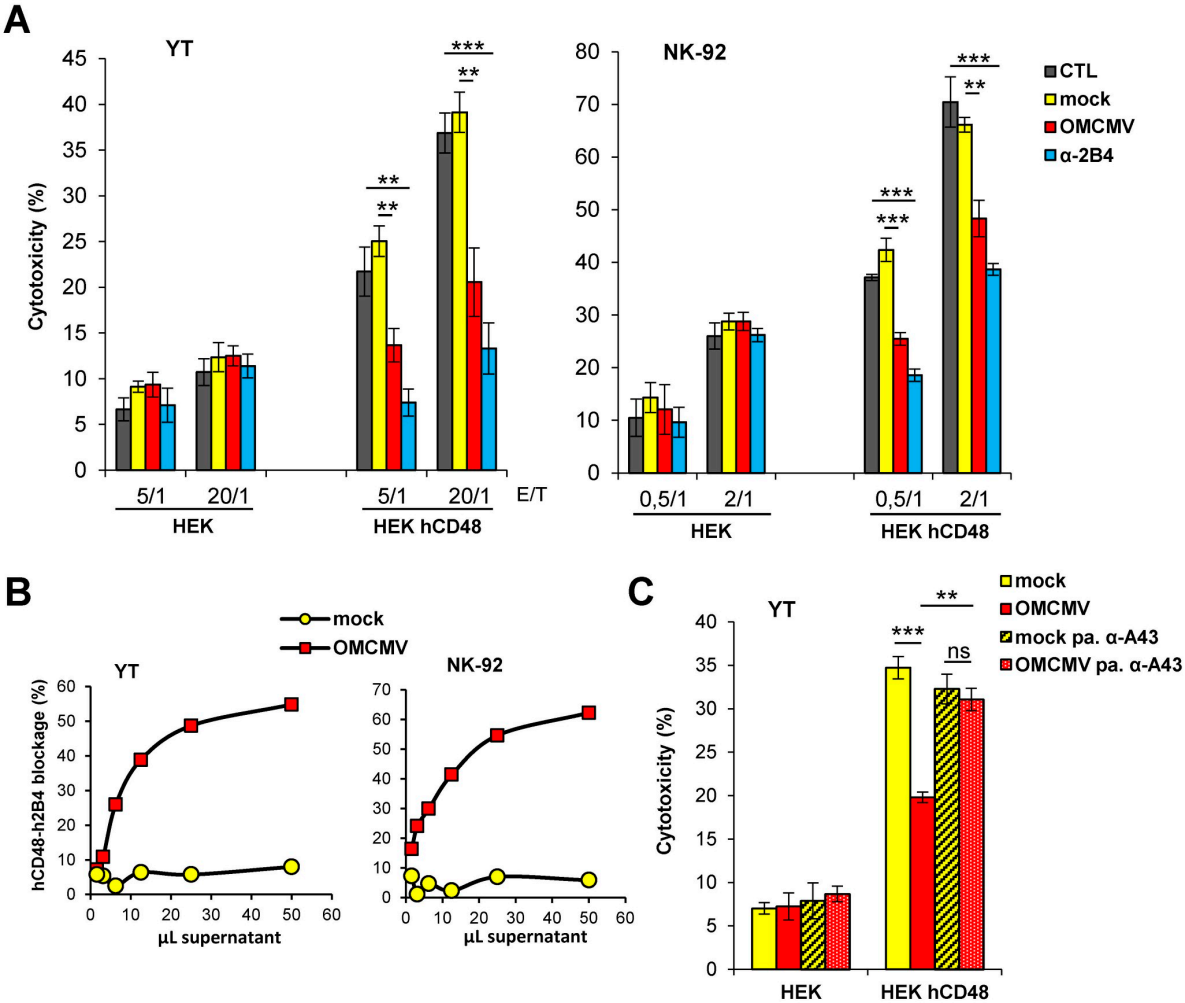
A43 secreted from OMCMV infected cells blocks NK cell cytotoxicity. (A) Same as in Fig 9A, except that YT or NK-92 cells were pre-incubated for 30 min with 50 μL of supernatants from OMK cells mock infected or infected with OMCMV diluted 1/2, with 10 μg/mL of α-2B4 mAb, or left untreated (CTL). (B) Flow cytometry analysis of YT or NK-92 cells exposed to different volumes of supernatants derived from OMK cells either mock infected or infected with OMCMV (moi of 2 for 6 days), followed by biotinylated human CD48-Fc. One representative experiment of each assay is shown. Percentages of blockade of hCD48-Fc cell binding were calculated from the MFIs obtained. (C) Same as in A at a 20/1 E/T ratio, except that YT cells were pre-incubated for 30 min with 50 μL of supernatants from OMK cells mock infected or infected with OMCMV diluted 1/2, or with these supernatants pre-absorbed (pa.) with the anti-A43 polyclonal antibody. The asterisks indicate that the difference between both points is statistically significant. ** p <0.01, *** p <0.001.

Lastly, we explored whether the viral A43 protein secreted during infection could also interfere with NK cell-mediated lysis. To this end, we measured YT or NK-92 cell-mediated cytotoxicity against hCD48-expressing HEK cells in the presence of the supernatant from OMCMV infected cells, using the supernatant from mock infected cultures as controls. As shown in Fig 12A, the supernatant from the OMK infected cells, but not from OMK mock-infected cells, led to a marked reduction of the 2B4-dependent YT and NK-92 cell-mediated killing at the two different E/T ratios evaluated. These observations were in corcordance with the specific ability of the infected culture supernatant to substantially inhibit the binding of hCD48-Fc to the two NK cell lines, in particular at the highest concentration evaluated, which was the one used in the cytotoxicity assay (Fig 12B). Furthermore, to demonstrate that the viral A43 protein present in the supernatant fom the OMCMV infected cells was responsible of the blockade of the h2B4-dependent NK cell cytotoxicity, we depleted A43 from the supernatant of the infected cultures by pre-adsorbing it with the anti-A43 polyclonal antibody attached to beads. As shown in Fig 12C, the supernatant from OMK infected cells depleted of A43 was not longer capable to decrease the h2B4-dependent YT cell-mediated killing. Thus, these results demonstrate that the A43 produced during infection is capable to impair NK cell cytotoxicicty.

## Discussion

Acquisition of host defense genes via horizontal gene transfer constitutes an important process by which CMVs develop new immunomodulatory strategies [28–30]. The existence of different versions of several SLAM family receptors, including *CD48*, among CMV genomes indicates that the targeting of SLAM-mediated immune responses is evolutionarily advantageous for these pathogens [22, 23]. Here, we have performed an in-depth functional analysis of the OMCMV encoded CD48 homolog A43, showing that it serves as a decoy molecule blocking 2B4-mediated NK cell cytotoxic responses.

Our assays demonstrate that *A43* is an immediate early gene that gives rise to a protein released from the infected cells within hours after infection. SPR assays indicated that soluble A43 binds to host 2B4 with high affinity and stability, exhibiting a K_D_ of 1.3 nM, in a comparable way than its natural ligand (K_D_ of 3.3 nM). In this sense, we must take into account that the N-terminal IgV-like domains of host CD48 and A43 are highly conserved (91% amino acid identity). All consensus residues of Ig superfamily members and those characteristic of SLAM family receptors are preserved in A43. Moreover, only one of the 14 amino acids of CD48 involved in the CD48:2B4 interaction [31] has been substituted by a different residue in A43. This is especially relevant since it has been estimated that the CD48 gene from which A43 derives was captured 19 million years ago [23]. This extraordinary degree of sequence preservation of the Ig domain, which contrasts with the substantial divergence of the rest of the A43 molecule, indicates a strong evolutionary pressure for mantaining its ligand binding specificity and affinity, in order to retain the properties most beneficial for viral survival.

We show that both, recombinant A43-Fc as well as the viral protein shed to the medium of tranfected or infected cells, prevent host CD48:2B4 interactions. Moreover, we present evidences that purified A43 can substantially reduce the interaction of host 2B4-transfected cells and host B lymphocytes that endogenously express CD48. Altogether, and taking into consideration the soluble nature of A43, these findings indicate that the viral protein serves as a decoy molecule, establishing a stable, long-lasting complex with 2B4 that prevents cytotoxic cells from recognizing its counter receptor CD48 on the surface of infected cells. Mammalian decoy receptors are soluble versions of cell surface receptors structurally incapable of signaling. They are able to down-modulate key biological functions by binding with high affinity and specificity to their ligands [32]. Viruses, such as poxviruses and herpesviruses, encode in their genomes a number of secreted decoy receptors that target mainly cytokines and chemokines [33–35]. The function of A43 described here illustrates that secreted viral decoys can also target additional key molecules involved in the antiviral response.

Importantly, we report that A43 is also capable of binding to human 2B4. This is remarkable since the N-terminal Ig domains of human and host CD48 share only 63% identity. Indeed, we show that the purified A43-Fc fusion protein specifically binds to h2B4-transfected COS-7 cells, as well as to human NK and T cell lines that endogenously express h2B4. In addition, SRP assays revealed that soluble A43 interacts with surface-immobilized h2B4 with high affinity (K_D_ 3.6 nM), in a similar way that it is observed for the interaction of the viral protein to host 2B4. However, in this case, when compared with hCD48, A43 was found to bind with a higher affinity (around 6-fold) to h2B4. Moreover, of particular relevance is the difference in the dissociation rates between A43:h2B4 and hCD48:h2B4 interactions, with A43 dissociating from h2B4 much slower than hCD48 (55-times). It should be pointed out, that the affinities reported here for hCD48:h2B4 interactions diverge with preexisting affinity data, which were in the low μM range [4]. This discrepancy may be due to differences in the experimental conditions and/or in the biosensor platforms used.

Due to the impossibility of obtaining NK cells from the OMCMV host, the owl monkey *A trivirgatus*, the finding that A43 is capable of binding to human NK cells allowed us to assess A43 at the functional level. NK cell cytolytic responses against virally infected cells are determined by the balance of inhibitory and activating signaling pathways, with the 2B4 receptor contributing to this process [36, 37]. Virtually all cytolytic NK cell responses depend on several coordinated steps, requiring a very first stage of contact between the NK cell and the potential target cell, with the implication of integrins, cytoskeletal proteins as well as NK cell receptors, followed by the formation of the immune synapse, sustained signaling, and directed delivery of lytic granules onto the target cell [38]. Engagement of 2B4 by CD48 on the NK cell leads to the initial scanning of target cells and the synapse formation, inducing its recruitment and clustering into lipid rafts [26]. Subsequently, the phosphorylation of the ITSMs in the 2B4 cytoplasmic tail induces its association with the adaptor molecule SAP, which enhances NK cytotoxicity [9, 39–41]. Here, we explored the function of A43 by employing a number of different assays. We provide evidence that NK cell-mediated cytotoxicity against hCD48 expressing target cells is pronouncedly impeded when NK-92, YT cells or primary human NK cells are incubated with soluble purified A43 or supernatants from infected cells. In addition, we show that in the presence of A43, NK cell production of IFN-γ is severely impaired. Our data also indicate that by masking h2B4 in NK cells, the viral protein disrupts the formation of conjugates and the establishment of the mature immunological synapse between effector and target cells. Lastly, we also present data that demonstrate that soluble A43 can interfere with human NK cell-mediated lysis of host B lymphocytes, which endogenously express CD48, or host CD48-expressing HEK cells. Taking into account the IE kinetics of A43, we propose that this viral protein may provide a mechanism for rapid counteraction of innate responses soon after infection, reducing host control of viral replication.

The results obtained here further emphasize the important function of the CD48/2B4 axes in the antiviral response. We must consider that in addition to OMCMV, other CMVs and large DNA viruses encode vCD48s, and therefore have the potential to act as CD48 decoys or to block 2B4 responses in alternative ways. Interestingly, some E3 proteins of human adenoviruses, reported to share a very low homology with SLAM receptors, show a promiscuous binding to several cell-surface molecules, including 2B4 [22, 42]. In addition, viral proteins can have the ability to interfere the CD48:2B4 axis by different mechanisms without interacting with 2B4; for example, by downregulating CD48 at the surface of the infected cell. Indeed, we have reported that MCMV, which encodes the early m154 protein responsible for driving proteolytic degradation of CD48, can lead to defective NK cell responses during infection [21]. Consequently, an MCMV mutant lacking m154 results in an attenuated phenotype *in vivo*, which can be substantially restored after NK cell depletion in mice. Moreover, human CMV (HCMV) also uses in part this strategy, reducing CD48 surface levels on human macrophages, although in this case the viral protein causing these effects is not yet known [43]. However, it must be noted that HCMV does not completely eliminate CD48 from the surface of infected cells, and consistent with this, blockade of 2B4 with a mAb inhibits only partially the human primary NK cell degranulation triggered by the infected macrophages. While HCMV does not encode a recognizable CD48 homolog, we cannot discard that it expresses a soluble protein that, in a similar way to A43, could contribute to block NK cell functions. This might not be surprising, taking into account that CMVs use diverse and redundant strategies to interfere with some of their immune targets. In this context, it seems that viruses have adopted different, and perhaps complimentary, tactics to modulate CD48:2B4 mediated effects.

Given that 2B4 is expressed on other immune cytotoxic cells, such as CD8^+^ T cells, it is more than likely that A43 also has the capacity to interfere with their functions via a similar blocking mechanism to that reported here for NK cells. Thus, expression of A43 could be of particular importance for the viral evasion of both innate and adaptive immune surveillance.

Interestingly, CD48 has also been identified as a soluble form in human serum, and shown to be at increased levels in patients with arthritis, mild asthma, and advanced lymphoid malignancies, potentially acting as an antagonist or decoy receptor capable of blocking CD48:2B4 binding [44–46]. Thus, considering the exceptional binding kinetic features of A43 compared to human CD48, one key aspect derived from this work is the potential of using A43 to develop novel therapeutic tools to manipulate aberrant immune responses, such as autoimmune diseases.

In summary, our work contributes a novel example of the potency of viral proteins to achieve immune modulation, in this case by counteracting the CD48/2B4 axis through a previously undescribed mechanism involving the direct blockade of 2B4.

## Material and methods

### Ethics statement

All procedures involving animals and their care were approved (protocol number CEEA 308/12) by the Ethics Committee of the University of Barcelona (Spain) and were conducted in compliance with institutional guidelines as well as with national (Generalitat de Catalunya decree 214/1997, DOGC 2450) and international (Guide for the Care and Use of Laboratory Animals, National Institutes of Health, 85–23, 1985) laws and policies. Human blood was obtained from healthy volunteer donors through the Blood and Tissue Bank of the Catalan Department of Health (Barcelona, Spain). Utilization of blood products for the experiments conducted was approved by the Ethics Committee of the Hospital Clinic of Barcelona (Barcelona, Spain), and according to the principles of the Declaration of Helsinki.

### Cell culture and viral infections

The cell lines COS-7 (green monkey fibroblast), HEK-293T (human embryonic kidney), NS-1 (mouse myeloma), YT (human NK), HSB2 (T lymphocyte), and MOLT4 (T lymphocyte) were obtained from the American Type Culture Collection. The owl monkey kidney cell line OMK (637–69) was from Sigma-Aldrich, and the human NK-92 cell line was kindly provided by T. Bellón (La Paz University Hospital Health Research Institute, Madrid, Spain). COS-7 and HEK-293T cells were cultured in Dulbecco’s modified Eagle’s medium supplemented with 2 mM glutamine, 1 mM sodium pyruvate, 50 U of penicillin per ml, 50 g of streptomycin per ml, and 10% fetal bovine serum. NS-1, YT, HSB2, and MOLT4 cells were cultured in RPMI-1640 medium, and OMK and NK-92 cells in Alpha Minimum Essential medium, supplemented as indicated above. In addition, 12.5% horse serum and 200 U/mL of rhIL-2 (Immunotools) were added to the medium of the NK-92 cells. B lymphocytes from *A*. *trivirgatus* owl monkeys were immortalized by infection with Epstein-Barr virus (EBV; provided by Montse Plana [Institut d’Investigacions Biomèdiques August Pi i Sunyer, Barcelona, Spain)]), following standard protocols. Briefly, fresh blood samples from *A*. *trivirgatus* were obtained from Faunia (Madrid, Spain). Erythrocytes were lysed by hypotonic buffer for 15 min, and 1x10^6^ cells were resuspended in 500 μL of RPMI-1640 medium supplemented as indicated above, with the addition of 20% of fetal bovine serum and 1μg/mL cyclosporin A (Sigma-Aldrich). Then, cells were mixed with 500 μL of supernatant from the marmoset B cell line (B95-8) that produces EBV, incubated overnight at 37 °C, washed, and grown during 4 weeks to obtain the *A*. *trivirgatus* polyclonal B lymphocyte cell line. Human PBMCs were isolated by Ficoll density-gradient centrifugation from fresh blood samples obtained from healthy human donors, as described previously [47]. Human NK cells were isolated from PBMCs by negative selection using the human NK Cell Isolation kit (Miltenyi Biotec) according to the manufacturer’s instructions by employing an autoMACS column on the autoMACS Pro separator (both Miltenyi Biotec). The efficiency of the process was confirmed by flow cytometry with anti-human CD56, CD16, and CD3 antibodies. The owl monkey cytomegalovirus (OMCMV) was provided by A. Davison (Medical Research Council, University of Glasgow Center Virus Research, Glasgow, United Kingdom; [48]). Viral stocks were prepared by infecting at a low moi OMK cells with OMCMV. Cell supernatants were recovered when maximum cytopathic effect was reached, and then cleared of cellular debris by centrifugation at 1700 g for 10 min. Viral titers were determined by standard plaque assays on OMK cells. Except for viral stock preparations, infections included a centrifugal-enhancement-of-infectivity step [49]. The supernatants from mock-infected or infected cells used in blocking and cytotoxicity assays were further clarified by ultracentrifugation at 34500 g for 90 min to remove viral particles and concentrated 20-fold using the Amicon Ultra-15 Centrifugal Filter Unit with an Ultracel-30 membrane (Millipore).

### Antibodies

The anti-human 2B4 (clone 2B4.69), anti-HA (employed for flow cytometry), and anti-human IgG (clone 29.5; Fc specific) mAbs were generated in our lab and have been previously described [23, 50]. The anti-human CD48 (99A) was provided by R. Vilella (Hospital Clinic, Barcelona, Spain). The rabbit anti-HA (clone c2974) used for western ELISA and the anti-perforin mAb employed for immunofluorescence were purchased from Cell Signaling MP, Biomedicals, and Immunotools, respectively. The anti-human CD3-FITC (clone 17A2), CD16-Brilliant Violet 421 (clone 3G8), and CD56-PerCP-Cy5.5 (clone B159) used to phenotype primary NK cells were obtained from BioLegend and BD Biosciences. The anti-human IgG-POD (Fc specific) and the streptavidin-POD conjugate were from Roche. The anti-mouse IgG-PE was purchased from Jackson ImmunoResearch, the goat anti-mouse IgG (H+L)-Alexa Fluor 555 was from Life Technologies, and the streptavidin-PE conjugate from BD Biosciences. The polyclonal Ab against A43 was obtained from two BALB/c mouse immunized four times with the A43-Fc fusion protein. Mice were bled, serum collected, and the mice IgGs purified using an Affi-Gel Protein-A MAPS II kit (Bio-Rad). The anti-A43 antibody coupled to protein G Sepharose resin (GE Healthcare) was used to pre-absorb (for 1h at room temperature in a rotating shake) the A43 protein present in the OMCMV infected supernatants. When required, purified antibiodies were biotinylated using biotinamidocaproate N-hydroxysuccinimide ester (Sigma-Aldrich).

### Plasmid constructions

HA-CD48-Tm, containing the N-terminal HA-tagged ectodomain of *A*. *trivirgatus* CD48 without its signal peptide fused to the platelet-derived growth factor receptor (PDGFR) transmembrane domain (Tm), was constructed as follows: first, PCR products were generated using as template DNA extracted from OMK cells and primer sets based on regions flanking the second and third exons of *Callihtrix jacchus* and *Saimiri boliviensis* CD48s, corresponding to the first and second Ig domains, respectively. The transcript annotations of CD48 considered were those of GenBank XP_008982993.1 of *C*. *jacchus* and GenBank XP_003938006.1 of *S*. *boliviensis*. The resulting PCR products were inserted into the pGEM-T vector (Promega) and sequenced. The newly identified nucleotide sequence was deposited in GenBank under the following accession number: MH663530 (exon2 and exon3 of *A*. *trivirgatus* CD48). Splicing by overlap extension (SOE)-PCR was then performed to join sequences coding for the first and second Ig domains of this molecule. The generated plasmids were used as templates and, based on their sequencing, two internal sequence-complementary primers, annealing within the Ig domains, and two external primers were employed for PCR amplification. These last two primers contained restriction sites at 5’ and 3’ ends, which were subsequently used to clone the SOE-PCR amplified products in the pDisplay vector (Invitrogen). HA-A43, HA-A43-Tm, *A*. *trivirgatus* 2B4-Fc (2B4-Fc, named aoCD244-Fc before), full-length human CD48 (hCD48), and human 2B4 (h2B4) were previously described [23, 50]. HA-2B4, expressing the N-terminal HA-tagged ectodomain of *A*. *trivirgatus* 2B4 fused to a region of human 2B4 (from residue 219 in the stalk segment to stop codon), was constructed by generating two independent PCR products that were subsequently linked making use of a natural *Sal*I restriction site present in the stalk region of both 2B4s. The first amplified fragment, containing the *A*. *Trivirgatus* 2B4 ectodomain was obtained using 2B4-Fc as template and a specific primer set, with the reverse primer containing the *Sal*I restriction site at the 3´end. The second PCR product, encompassing the stalk, Tm and cytoplasmic regions of human 2B4 was generated using the h2B4 construct as template and a specific primer set, with the forward primer containing the *Sal*I restriction site at the 5´end. These PCR products were independently cloned into pGEM-T, and then inserted consecutively into the pDisplay vector, using the shared *Sal*I restriction site to join both fragments. HA-A43 ΔG-R and HA-A43 ΔT-R expressing full length HA-A43 with a deletion in residues 214–219 (HA-A43 ΔG-R) or 220–225 (HA-A43 ΔT-R), and HA-A43 K216P and HA-A43 K216A with the lysine in position 216 exchanged by proline (K216P) or alanine (K216A), were constructed by SOE-PCR as described above, using HA-A43 as a template and internal primers bearing the corresponding deletions or mutations. PCR-amplified products were inserted into the pGEM-T vector and transferred to pDisplay. A43-Fc, *A*. *trivirgatus* CD48-Fc (CD48-Fc), hCD48-Fc and h2B4-Fc fusion proteins, expressing the two Ig domains of these molecules (with the CD33 leader peptide replacing their own signal peptide) fused to the Fc region of human IgG_1_, were obtained by PCR using as templates HA-A43 or HA-CD48-Tm and hCD48 or h2B4 constructs, respectively, and specific set of primers with restriction sites. The PCR-amplified products were inserted into pGEM-T and finally cloned into pCI-neo Fc vector, as described before [23]. A mutant version of the Fc region of human IgG_1_, named here Fc*, containing mutations L234F, S235Q, K322Q, M252Y, S254T, and T256E, was obtained by chemical synthesis (Genscript). The mutations introduced abrogate the binding of the Fc region to Fc receptors [27]. pCI-neo A43-Fc*, expressing the two Ig domains of A43 fused to the Fc*, was obtained by replacing the Fc region present in plasmid A43-Fc by the Fc* region. All PCR reactions were performed under the following conditions: 1 cycle at 94°C for 5 min; 30 cycles of 1 min at 94°C, 1 min at 51°C, and 1 min at 72°C; and 1 cycle at 72°C for 10 min. For the annealing reactions, the conditions were: 6 cycles of 5 min at 94 °C; 1 cycle at 51 °C for 1 min; 1 cycle at 72 °C for 1 min; and 1 cycle of 10 min at 72 °C. The identification of all recombinant plasmids was confirmed by DNA sequencing.

### RT-PCR

OMK cells were mock infected or infected with OMCMV at an moi of 1. The inhibitory chemicals CHX (100 μg/ml; Sigma-Aldrich) or PAA (250 μg/ml; Sigma-Aldrich) were added to some cultures to assess selective expression of viral immediate-early genes or early genes, respectively. The cultures were treated with CHX 30 min prior to infection, whereas phosphonoacetic acid was added at the time of infection, and both inhibitors were maintained until RNA was harvested. Total RNA was isolated at different time points after infection (13 hours post infection for CHX samples and 72 hours post infection for the rest) by the TRIzol method (Invitrogen). RT-PCR was then carried out using the SuperScript III First-strand Synthesis System for RT-PCR (Invitrogen) according to the manufacturer’s protocol. Briefly, RNA samples were treated with RNase-free DNase I (Promega) for 30 min at 37°C, and the DNase was inactivated at 65°C for 10 min. The RNA was reverse transcribed using oligo(dT) primers at 50°C for 50 min, and reactions were terminated by heating at 85°C for 5 min. The reverse-transcribed products were treated with RNase H for 20 min at 37°C and amplified by PCR as indicated above using specific primer sets. Amplified products (a 599-bp fragment for *A43*; a 590-bp fragment for OMCMV *IE1*; a 570-bp fragment for OMCMV *UL54*; a 166-bp fragment for OMCMV *UL73*; and a 101-bp fragment for *GAPDH*) were separated on a 1% agarose gel and visualized by RedSafe nucleic acid staining solution (iNtRON Biotechnology Inc.).

### Transfections and generation of Ig fusion proteins

COS-7 cells were transiently transfected with 5 μg of the indicated plasmid using the Amaxa Cell Line Nucleofector Kit R according to the manufacturer’s protocol. HEK cells were transiently transfected with 0.2 μg/cm^2^ of the indicated plasmid mixed with 6 μL/μg DNA of polyethylenimine (1mg/mL, Sigma-Aldrich) in 0.1 mL/cm^2^ of OPTIMEM medium (Gibco) for four hours. Then cultures were washed and used either one day later when performing functional assays or 6 days later to collect A43-Fc, hCD48-Fc, CD48-Fc or host 2B4-Fc fusion proteins from supernatants. The NS-1 stable transfectant secreting m2B4-Fc fusion protein has been previously described [21]. The NS-1 h2B4-Fc stable cell line was obtained by the same procedures. Briefly, eight million of NS-1 cells were electroporated (280V, 950 mF) with 8 μg of linearized DNA using the Gene Pulser II Apparatus (Bio-Rad), selected with 1.2 mg/mL of geneticin (G418, Invitrogen), and further subcloned. The clone producing the highest amounts of fusion protein was cultured in INTEGRA CL 1000 flasks (Integra Biosciences AG). The supernatants containing the fusion proteins were purified using the Affi-Gel Protein-A MAPS II kit (Bio-Rad). To analyse A43 shedding, supernatants of COS-7 cells transfected with HA-A43 or HA-A43 K216P were used after being cleared to remove cellular debris. HA-A43 supernatants employed in blocking experiments were further concentrated 20-fold as indicated above for the supernatants of infected cells.

### ELISA

Detection of the HA-A43 protein in cell culture supernatants was performed by sandwich ELISA using the rabbit anti-HA mAb to coat the ELISA plates and the biotinylated anti-A43 polyclonal antibody, followed by incubation with streptavidin-POD conjugate. A similar sandwich ELISA was performed to detect A43 in the supernatants from infected cells, except that in this case the anti-A43 polyclonal antibody was also used as the capture antibody. The capacity of the A43 protein to block host 2B4:CD48 or h2B4:hCD48 interactions was assessed by sandwich ELISA using host 2B4-Fc or h2B4-Fc coated ELISA plates and biotinylated host CD48-Fc or hCD48-Fc for detection, respectively, followed by streptavidin-POD. The concentration of IFN-γ in the supernatants of the co-cultures of NK cells and HEK cells non-transfected or transfected with hCD48, was measured using the high-sensitivity human ELISA set (ImmunoTools). ELISA measurements of Optical densities (ODs) were done at 450 and 570 nm (Thermo Scientific Multiskan FC).

### Flow-cytometry analysis

Flow cytometry was performed using standard procedures [51]. To minimize non-specific staining, all incubations were carried out in the presence of 20% rabbit serum (Linus) and 1% of fetal bovine serum in PBS. Cells were stained with the corresponding antibodies, followed by anti-mouse IgG-PE. Fc fusion protein stainings were performed using the indicated amount of the Fc fusion protein followed by incubation with the anti-human IgG (Fc specific) and by anti-mouse IgG-PE. An irrelevant Fc fusion protein (CTL-Fc) was always used as a negative control. When performing assays to block the interaction of hCD48 and h2B4, cells were incubated with biotinylated hCD48-Fc fusion protein, followed by streptavidin-PE.

### SPR kinetic affinity measurement

SPR was performed on a Biacore X100 instrument (GE Healthcare) as described [52]. Recombinant host 2B4-Fc or h2B4-Fc proteins were immobilized at low density on CM4 chips by amine coupling. Increasing concentrations of host CD48, hCD48 or A43 recombinant proteins were injected in HBS-EP buffer (10 mM HEPES, 150 mM NaCl, 3 mM EDTA, 0.005% [vol/vol] surfactant P20 [pH 7.4]) at 30 μl/min during 2 min and a 5-min dissociation was recorded. A 10–1500 nM concentration range of analyte was used. Between analyte injections, surface was regenerated with 10 mM glycine-HCl pH 2.0. Kinetic data were globally fitted to a 1:1 Langmuir model using the Biacore X100 Evaluation software version 2.0.1 (GE Healthcare). Bulk refractive index changes were removed by subtracting the responses recorded in the reference flow cell, and the response of a buffer injection was subtracted from all sensorgrams to remove systematic artifacts.

### Adhesion assay

COS-7 cells, transiently transfected with host HA-2B4 or hCD48, or the corresponding empty plasmids, were cultured on glass coverslips in 24-well tissue culture plates. After 24 hours, cells were washed with PBS and maintained at 4°C for 20 min. *A*. *trivirgatus* B lymphocytes or NK-92 cells were added to wells for 1 hour at 4°C at a concentration of 1x10^6^ or 5x10^5^ cells/well, respectively. Subsequently, coverslips were washed by repeated immersion (20X) in culture medium, and the adhesion of *A*. *trivirgatus* B lymphocytes or NK-92 cells to the COS-7 transfected cells was examined using an inverted Leica DMI600 B microscope (Leica microsystems), where contrast images were obtained. When indicated, adhesion assays were performed with *A*. *trivirgatus* B lymphocytes or NK-92 cells previously stained with calcein-AM (4 μM, Life Technologies) for 30 min, followed by two washing steps, as previously described [53]. Cells were trypsinized and analyzed by flow cytometry using a FACSCalibur flow cytometer (BD Biosciences) and FlowJo software (TreeStar Inc). Cells were gated based on forward and side scatter, examined for calcein labelling, and the percentages of calcein stained *A*. *trivirgatus* B lymphocytes or NK-92 cells versus unlabelled COS-7 cells were calculated. Cellular adhesion-blocking experiments were performed by pre-incubation of COS-7 HA-2B4 or NK-92 cells with 10 μg/mL of A43-Fc, an unrelated CTL-Fc protein, and in the case of NK-92 cells with the anti-human 2B4 mAb or an isotype control antibody, for 30 min at 37°C.

### Conjugate formation assay

NK-92 and YT cells were labelled with CellTracker Blue CMAC (10 μM, Invitrogen) for 30 min at 37°C. HEK cells, transfected with hCD48 or the corresponding empty vector, were stained with CellTrace CFSE (0.5 μM, Invitrogen) for 15 min at 37°C according to the manufacturer´s instructions. After labelling, cells were washed extensively. Then, 1x10^5^ NK-92 or YT cells were co-cultured at 37°C for 0 or 10 min with HEK cells at a ratio of 1/1, in a final volume of 150 μL. Reactions were stopped by adding 50 μL of ice-cold PBS. Conjugates were detected by flow cytometry as double positive events. When indicated, labelled NK cells were pre-incubated with 10 μg/mL of A43-Fc, an unrelated CTL-Fc protein or anti-2B4 mAb, at 37°C for 30 min.

### Immunological synapse observation by immunofluorescence microscopy

HEK cells transfected with hCD48 or the corresponding empty vector were first labelled with CellTracker Blue CMAC. After washing, labelled HEK cells were mixed for 10 min at 37°C and a final volume of 500 μL with 1x10^5^ YT cells at a ratio of 1:1 to allow conjugate formation. The suspension was placed on coverslips coated with poly-L-lysine (Sigma-Aldrich) for 10 min at 37°C, washed with PBS, fixed with 4% formaldehyde for 10 min, and permeabilized with 0.05% Triton X-100 for another 10 min. Subsequently, samples were blocked with 20% rabbit serum and 6% fetal bovine serum in PBS and incubated with Alexa Fluor 488 Phalloidin (Invitrogen) and the primary anti-perforin mAb, followed by a secondary antibody goat anti-mouse IgG (H+L)-Alexa Fluor 555. Coverslips were mounted in ProLong Gold antifade reagent (Invitrogen). Fluorescence images were acquired using a Nikon Optiphot-2 microscope (Nikon Corp.). Synapse stages were defined as: 0, conjugates lacking actin polymerization and perforin polarization; 1, conjugates with polymerized actin, but missing perforin polarization; 2, conjugates with actin polymerization and perforin clustered at the immune synapse. When indicated, NK cells were pre-incubated with 10 μg/mL of A43-Fc or CTL-Fc fusion proteins, at 37°C during 30 min prior to being mixed with the target cells.

### NK cell cytotoxicity assay

The cytotoxic activity of NK-92 or YT effector cells was determined in a calcein-AM release assay [54]. The assay was performed in V bottom 96-well microtiter plates. Briefly, transfected target HEK cells or *A*. *trivirgatus* B lymphocytes were labelled with calcein (4 μM) for 30 min at 37°C. After two washes, 2x10^4^ target labelled cells per well were mixed at 37°C with effector cells at E/T ratios ranging from 20/1 to 0.5/1, in triplicate. The assay also included six replicate wells with only target cells (spontaneous release), only target cells lysed with the addition of 1% Triton X-100 (maximum release), or medium alone (background). Four hours later, 100 uL of supernatant of each culture was collected and transferred into new plates. When indicated, effector cells were pre-incubated with 10 μg/mL of A43-Fc or A43-Fc*, unrelated CTL-Fc or CTL-Fc* proteins, anti-2B4 mAb or an isotype control antibody, or supernatants from mock-infected or OMCMV infected cells, for 30 min at 37°C. Fluorescence was measured using Spark multimode microplate reader (Tecan). The background fluorescence was substracted from all samples. The percentage of specific lysis was calculated according to the formula: (experimental release—spontaneous release) / (maximum release—spontaneous release) x 100.

### Protein domain prediction

Signal peptides and transmembrane regions were predicted by using SignalP 4.1 and TMHMM 2.0, respectively. These bioinformatics prediction tools are available at http://www.cbs.dtu.dk/services/SignalP/ (SignalP 4.1), and http://www.cbs.dtu.dk/services/TMHMM-2.0/ (TMHMM 2.0). Ig domains were determined from annotations in CDD [55]. To calculate the percentage of amino acid identity of the N-terminal Ig domains of A43 and *A*. *trivirgatus* CD48, protein sequences were paired aligned (MAFFT version 7.402; [56]) and positions containing gaps were discarded.

### Statistical analyses

Analyses were performed with GraphPad Prism software (v.7.03) and Microsoft Excel (2010). Results are given as means ± standard deviations (SD) or as means ± standard errors of the means (SEM), and statistical significances were determined with the Student’s t-test (two-tailed). P-values less than or equal to 0.05 (*), 0.01 (**) and 0.001 (***) were considered statistically significant.
